# Enhancing intestinal barrier efficiency: A novel metabolic diseases therapy

**DOI:** 10.3389/fnut.2023.1120168

**Published:** 2023-03-02

**Authors:** Yaoyuan Zhang, Xiao Zhu, Xinyuan Yu, Petr Novák, Qingjun Gui, Kai Yin

**Affiliations:** ^1^Institute of Translational Medicine, Hengyang Medical School, University of South China, Hengyang, Hunan, China; ^2^Guangxi Key Laboratory of Diabetic Systems Medicine, Guilin Medical University, Guilin, China; ^3^Department of General Practice, The Fifth Affiliated Hospital of Southern Medical University, Guangzhou, Guangdong, China

**Keywords:** intestinal barrier, metabolic diseases, natural medicine, gut microbiota remodeling, lifestyle intervention, bariatric surgery

## Abstract

Physiologically, the intestinal barrier plays a crucial role in homeostasis and nutrient absorption and prevents pathogenic entry, harmful metabolites, and endotoxin absorption. Recent advances have highlighted the association between severely damaged intestinal barriers and diabetes, obesity, fatty liver, and cardiovascular diseases. Evidence indicates that an abated intestinal barrier leads to endotoxemia associated with systemic inflammation, insulin resistance, diabetes, and lipid accumulation, accelerating obesity and fatty liver diseases. Nonetheless, the specific mechanism of intestinal barrier damage and the effective improvement of the intestinal barrier remain to be explored. Here, we discuss the crosstalk between changes in the intestinal barrier and metabolic disease. This paper also highlights how to improve the gut barrier from the perspective of natural medicine, gut microbiota remodeling, lifestyle interventions, and bariatric surgery. Finally, potential challenges and prospects for the regulation of the gut barrier-metabolic disease axis are discussed, which may provide theoretical guidance for the treatment of metabolic diseases.

## 1. Introduction

Metabolic diseases, such as obesity, diabetes, hyperlipidaemia, and non-alcoholic fatty liver disease (NAFLD) have become widespread and significant public health problems (1). Specifically, the WHO released a report on the status of the obesity pandemic in Europe in May 2022, noting that 60% of citizens in the European region were overweight or obese, highlighting the impact of the obesity pandemic (2). The global prevalence of diabetes among individuals aged 20–79 was estimated to be 10.5% (536.6 million people) in 2021, and is expected to rise to 12.2% (783.2 million people) by 2045 (3). From 1991 to 2019, the estimated global prevalence of NAFLD increased sharply from 20 to 30% in the general population (4). Metabolic diseases are often caused by multiple factors, including excessive consumption of processed high-energy foods, lack of exercise, and environmental and genetic factors (5). However, current treatment strategies, including lifestyle changes, dietary and exercise interventions, and drug use, still have limited efficacy. Therefore, there is a need to identify reliable targets for the prevention and treatment of these diseases and their complications.

The intestinal barrier separates the human body from the intestinal microbes, viruses, food antigens, and environmental toxins. In healthy individuals, the intestinal barrier maintains normal gut microbiota and protects mucus layer physiological function (6) and balances epithelial cells and the gut immune system, which can maintain gut homeostasis and is crucial for the dynamic balance of the body (7). However, a significant increase in intestinal permeability has been observed in patients with obesity, NAFLD, and diabetes. The underlying mechanisms may be related to harmful changes in gut pathogenic bacteria and their products, which further increase barrier permeability. Host metabolic states, such as hyperglycaemia and hyperlipidaemia, have also been confirmed to decrease tight junction protein expression and disturb epithelial cell integrity. Both are considered crucial factors for intestinal barrier integrity (6, 8, 9). An impaired gut barrier leads to the translocation of microbiota-derived LPS into the circulatory system, and high circulating LPS levels, a condition referred to as metabolic endotoxemia is associated with obesity and related metabolic disorders (10). In addition, increases harmful gut bacteria, which can further aggravate gut infection and promote gut bacterial translocation to the blood and liver (11). Moreover, impaired gut barriers increase the transfer of intestinal-derived metabolites such as trimethylamine N-oxide, branched-chain amino acid, and indoxyl sulfate from the gut to the systemic circulation, which are also associated with the development of metabolic diseases (12, 13). Meanwhile, a compromised gut barrier can lead to the over-activation of the gut immune system, inducing chronic systemic inflammation or an impaired immune response, which promotes the progression of metabolic diseases (14).

In this review, we describe the critical molecular pathways and mechanisms underlying abnormal gut barrier function in metabolic diseases. Then, we summarize advances made in supporting the improvement of the intestinal barrier using natural medicines, gut microbiota remodeling, and lifestyle interventions. Lastly, we discuss the current challenges and prospects for treating metabolic diseases through the modulation of the intestinal barrier axis.

## 2. A short review of the intestinal barrier

The intestinal barrier is the primary defense against potentially harmful substances and pathogenic bacteria and consists of a physical barrier, a mucus barrier, and an immunological barrier (15–19). Intestinal physical barrier integrity is regulated by tight junctions and intestinal epithelial cell function. Tight junctions consist of transmembrane proteins such as claudin, occludin, zonula occludens-1 (ZO1), and cingulin between intestinal epithelial cells (20–22). The mucus layer is composed of many components: water, electrolytes, lipids, and about 30 proteins, most of which are produced by specialized secretory goblet cells (GCs), including mucin, human IgGFc-binding protein, calcium-activated chloride channel modulator 1, and zymogen granule protein 16. The mucus layer serves as a barrier covering the intestinal epithelium that prevents direct contact between antigens, toxins, gut flora, and epithelial cells while maintaining permeability to essential nutrients and macromolecules. In addition, at the same time, the outer mucus layer is used as the energy source of some bacteria to stabilize the balance of intestinal flora (23–25). The innate and adaptive immune systems in the intestinal tract are strictly regulated. The immune cells in the intestinal tract cooperate closely (macrophages, monocytes, neutrophils, dendritic cells, natural killer cells, eosinophils, and non-specifically recognized basophils) to achieve and maintain intestinal immune balance (26, 27). However, dietary disorders, diseases, and pressure affect the intestinal barrier function. Mechanically, intestinal barrier dysfunction is first caused by tight connection disorder, the loss of tight junctions causes intestinal mucus layer atrophy and secretory dysfunction (28), which in turn causes further immune cell activation by numerous antigenic molecules or microorganisms through the paracellular pathway, aggravating intestinal immune dysfunction (29).

## 3. The impaired intestinal barrier is a catalyst for the development of metabolic diseases

Intestinal barrier integrity is impaired in metabolic diseases, including diabetes, hyperlipidaemia, and cardiovascular diseases (30). Numerous studies have confirmed that metabolic disorders and diseases can cause harmful changes in the intestinal microenvironment, including abnormal lipid load, high glucose, high uric acid, and intestinal flora disturbances. This remodeling further damages the integrity of the intestinal barrier (31–33).

First, in the physical barrier section, metabolic diseases can impair the intestinal barrier by affecting the intestinal epithelial cell function and tight junction protein expression. High glucose levels can lead to abnormal intestinal epithelial cell function, abnormal lipids, and abnormal immune responses, and an intestinal microbiota imbalance can induce intestinal epithelial cell tight junction damage, further aggravating intestinal epithelial barrier dysfunction (34, 35). Additionally, metabolic diseases can contribute to abnormalities in the mucus barrier (36). Defects in the colonic mucus layer, characterized by increased permeability and reduced mucus growth rate, have been observed in obese mice. Moreover, the intestinal immune barrier is known to be impaired in metabolic diseases, wherein the main alteration is a decrease in the production of intestinal antimicrobial factors and the promotion of an increased release of pro-inflammatory cytokines, including IL-1b, IL-6, IL-12, and IL-18 (37).

**Figure 1:**
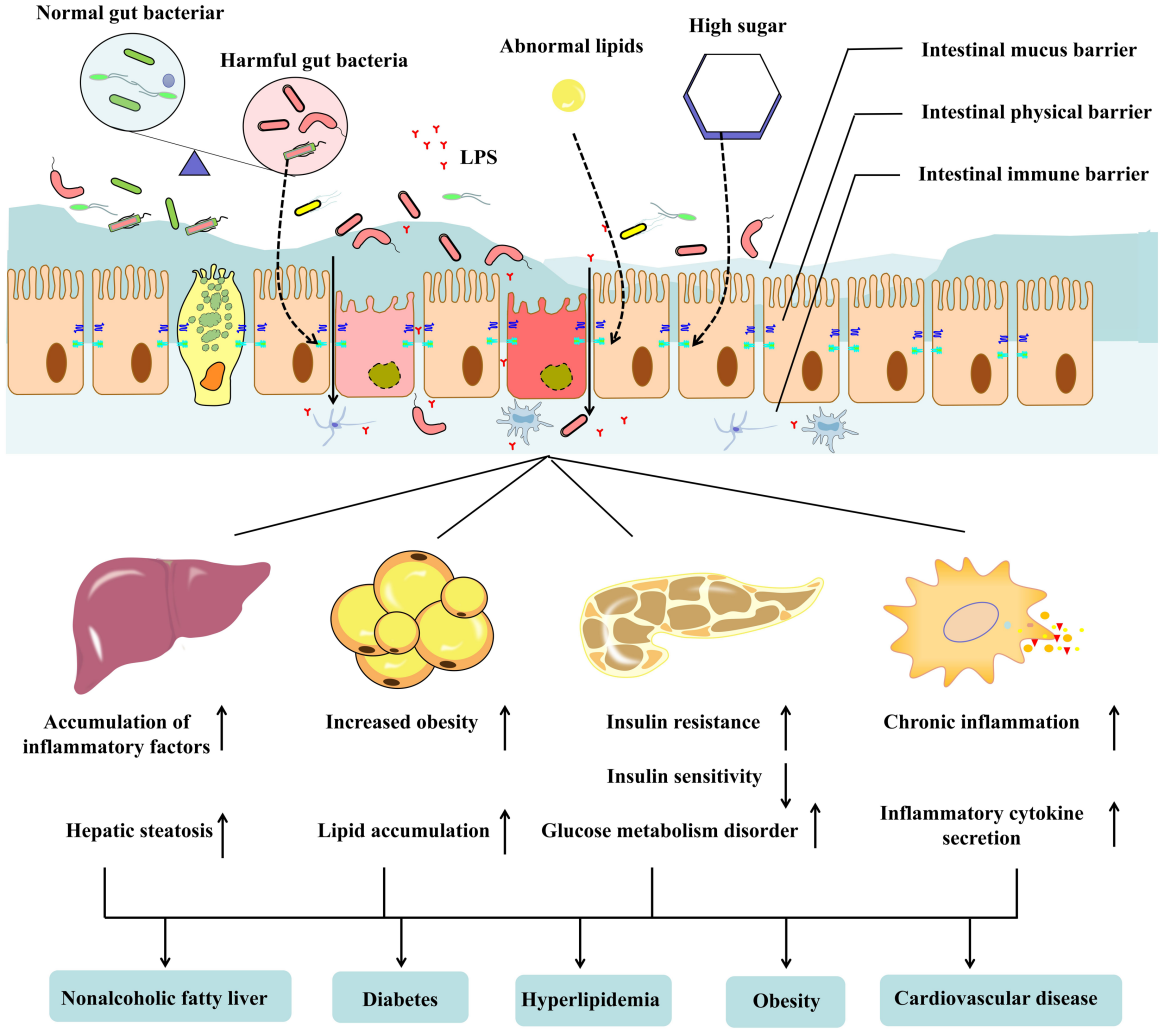
The gut barrier consists of gut commensal microbes, mucus, and immune cells in the intestinal epithelium and lamina. Metabolite disorders (hyperglycemia and abnormal lipids) and metabolic diseases, including obesity type 2 diabetes mellitus (T2DM) and non-alcoholic fatty liver disease (NAFLD), lead to gut dysbiosis and disrupt the integrity of the gut barrier. Harmful bacteria or lipopolysaccharides in the intestine enter the blood through the damaged intestinal barrier. It can affect the tissues and organs related to metabolic diseases, resulting in abnormal liver, adipose tissue, pancreas, and immune system functions, aggravating the occurrence and development of metabolic diseases further.

As an accelerator of metabolic diseases, the impairment of the intestinal barrier exacerbates systemic inflammation, impaired energy metabolism, insulin resistance, and abnormalities in glucose and lipid metabolism, thereby accelerating the progression of metabolic diseases (38). For instance, damage to the intestinal barrier can increase intestinal endotoxins in the blood, leading to chronic low-grade inflammation, further promoting the development of metabolic syndrome (39). In a population study, an increased intestinal permeability in pregnant women and an impaired intestinal barrier was found to lead to increased insulin resistance and decreased insulin sensitivity (40). A study of mice fed a high-fat diet found that intestinal barrier dysfunction leads to higher glucose metabolism disorders and liver steatosis (41, 42). Although the pathway by which an impaired intestinal barrier exacerbates metabolic disease has been partially demonstrated, the underlying mechanism still requires further exploration. With the development of high-throughput analysis, including serum and intestinal metabolomics and intestinal proteomics, the research depth and breadth of microbiota, intestinal barriers, and the host is likely to be further expanded in this future, and providing new directions for prevention and treatment metabolic diseases(Figure 1).

## 4. Improving the intestinal barrier as a target for the treatment of metabolic diseases

With advances in research, several strategies have been developed to improve the intestinal barrier in the treatment of metabolic diseases in rodent and clinical studies, including drug therapy, adjusting the composition of the intestinal flora, adding probiotics, exercise, diet, and bariatric surgery. In this section, we comprehensively summarize the experimental clinical studies on improving the intestinal barrier using these methods and explore the potential treatment mechanisms of metabolic disease (43–46).

### 4.1. Drugs improve gut barrier function in metabolic diseases

Currently, treatments for intestinal barrier damage are limited. Clinical drugs used to treat intestinal barriers include natural drugs, short-chain fatty acids, and those that improve intestinal inflammation (47). The main therapeutic targets of these drugs are increasing intestinal tight junction protein expression, improving intestinal cell function, and inhibiting intestinal inflammation. Studies have confirmed that in the clinical treatment of metabolic diseases, drugs such as metformin, berberine, and butyrate have been found to improve disease progression by modulating the intestinal barrier (48). Therefore, in this section, we mainly discuss the function and specific mechanisms of drugs, such as metformin, berberine, and butyrate, used to improve the intestinal barrier and provide new perspectives for treating metabolic diseases (Table 1).

**Table 1:** Drugs that improve the gut barrier.

Drug name	Source	Signal pathway	Effects	References
Metformin	Mice models	Activates the AMPK pathway	Increases ZO-1, occludin, and claudin-1 expression	(49)
	Mice models	AMPK1-dependent inhibition of JNK signaling activation	Increases ZO-1, occludin, and claudin-1 expression	(50)
	Mice models		Restores the tight junction protein occludin-1 levels	(51)
	Caco-2 cells	Inhibits the MLCK-MLC signaling pathway	Increases tight junction proteins	(52)
	Mice models	Inhibits Wnt signaling	Increases goblet cell mass and mucin production in the gut	(53)
	NAFLD mice		Mitigates the loss of tight junction proteins in the small intestine	(54)
	Caco-2 cells	Inhibits endoplasmic reticulum stress	Reduces intestinal epithelial cell apoptosis and increases tight junction protein expression	(55)
	IBS rats	Reduces PAR-2 expression inhibits ERK activation	Improves the interaction of clau-4 with ZO-1 and clau-4 expression	(56)
Butyrate	IEC cells	Promotes the interaction of transcription factor SP1 with the claudin-1 promoter	Increases claudin-1 transcription	(57)
	Diabetic mice		Increases intercellular adhesion molecules	(58, 59)
	Diabetic mice	Activated NLCR3 binds to GPR43 on colonic epithelial cells	Upregulates expression of the tight junction protein ZO-1/occludin	(60)
	H4 cell		Upregulates tight junction and mucus genes	(61)
	IPEC-J2 cells	Activates the Akt/mTOR-mediated protein synthesis machinery	Enhances the abundance of tight junction proteins	(18)
	IBD mice	Activates GPR109A and inhibits AKT and NF-κB p65 signaling	Improves intestinal epithelial barrier dysfunction	(62)
	CKD rats	Improves AMPK phosphorylation and increases GLP-1 secretion	Promotes colonic mucin and TJ proteins	(63)
Berberine	T2DM rat model		Improves the intestinal mucosa and immune barrier	(64)
	T2DM rat model		Increases ZO-1 expression and repairs damaged intestinal mucosa	(65)
	Caco-2 monolayers	Structural normalization and redistribution of the tight junction protein occludin	Improves intestinal epithelial tight junction injury	(66)
	HT-29/B6 human colon monolayers, rat	Mediated through the tyrosine kinase, pAkt, and NFκB pathways	Prevents the TNF-α-induced claudin-1 disassembly and upregulation of claudin-2	(67)
	T84 colonic epithelial cells	Ability to promote cell migration	Increased expression of intestinal tight junction and adhesion-linked proteins	(68)
	DSS-induced murine UC	Regulation of gut EGC-IEC-immune cell interactions	Improves mucosal inflammation	(69)
	DSS-induced murine UC	JAK–STAT pathway	Inhibits intestinal mucosal inflammation	(70)
	DSS-induced murine UC		Promotes anti-inflammatory and anti-oxidative stress responses	(71)
Infliximab	Crohn’s disease patients		Anti-inflammatory effects of TNF-α blockade	(72)
	Crohn’s disease patients	Normalization of apoptosis in colonic epithelial cells	Repairs the intestinal barrier	(73)
	Crohn’s disease patients		Improves intestinal mucosal barrier	(74)
	Crohn’s disease patients	Promotes Th22 cell differentiation and upregulates IL-22 production	Intestinal epithelial barrier repair	(75)
Oregonin	Caco-2 cells		Restores zonula occludens-1 and occludin expression	(76)
RIPK1 inhibitor	Intestinal epithelial cells	Inhibits necroptosis and the NF-κB signaling pathway	Reduces the disruption of tight junctions and accompanying oxidative stress	(77)
	Murine colitis model	FADD-RIPK1-caspase-3 signaling	Inhibits intestinal epithelial cell necrosis	(78)
Citrus nobiletin	Rat intestinal and Caco-2 cells	Inhibits the Akt-NF-κB-MLCK pathway	Restores damaged barrier function	(79)
Tofacitinib	Caco-2BBe intestinal epithelial cells		Improves intestinal epithelial-macrophage interactions	(80)
	Intestinal epithelial cells (IECs)		Restores claudin-2 expression levels	(6)

#### 4.1.1. Metformin

Metformin is an oral hypoglycaemic agent that is widely used as first-line treatment for type 2 diabetes. Metformin improves hyperglycaemia by inhibiting hepatic glucose production and increasing glucose uptake in muscles (81). Metformin has also been shown to reduce cardiovascular events and improve abnormal lipid metabolism and chronic inflammation (82). Furthermore, metformin has been found to improve the gut barrier, and its metabolic disease-treatment effect is partly based on modulating gut function (83).

Specifically, recent studies have confirmed that metformin can improve intestinal physical barrier function by increasing the expression of intestinal tight junction proteins (ZO-1, occludin, and claudin-1) by activating the AMPK pathway, reducing the entry of LPS into the blood and the inflammatory response to body stimuli (49–51). Cell experiments have also confirmed that metformin can stabilize and upregulate the expression of tight junction proteins by inhibiting the MLCK-MLC signaling pathway, thereby improving the tight junctions of intestinal epithelial cells (52). Moreover, metformin was found to improve the intestinal mucus barrier by beneficially regulating the quality of goblet cells and mucin production. It can be used to prevent and treat metabolic diseases in obese individuals and individuals on a western high-fat diet (53).

In addition, maintaining bile acid homeostasis can improve the intestinal barrier and prevent bacterial translocation in the intestinal tract, demonstrating efficacy in the treatment of metabolic liver disease (84). Interestingly, metformin can promote bile acid homeostasis in the liver and intestines. We speculated that metformin can target the homeostasis of the bile acid-intestinal barrier axis and be used to develop new methods for the treatment of metabolic diseases (85). Glucagon-like peptide-1 (GLP-1) is a peptide hormone in the gut that plays a central role in coordinating postprandial glucose homeostasis. The administration of GLP-1 and glucagon-like peptide-2 (GLP-2) receptor agonists promotes intestinal barrier function in mice (86). Interestingly, the insulin sensitiser metformin increased circulating GLP-1 concentrations and the relative number of intestinal L cells (87). Therefore, it is reasonable to believe that the improvement of the intestinal barrier by metformin is partly dependent on the regulation of GLP-1 (88). Notably, metformin concentrations are much higher in the gut than in the plasma. Although there is reason to believe that the maintenance of the gut barrier and gut axis plays a role in the efficacy of metformin, an understanding of the mechanisms by which metformin promotes a healthy gut barrier will require a systems-level approach.

#### 4.1.2. Butyrate

Butyrate is a four-carbon short-chain fatty acid fermented by the intestinal flora through dietary fiber (89). It meets most of the energy needs of colonic epithelial cells and is required for cellular energy metabolism and the maintenance of intestinal homeostasis. Butyrate supplementation has been investigated for its potential protective and ameliorative effects on a wide range of human diseases, including type 2 diabetes, cardiovascular disease, dyslipidaemia, and non-alcoholic fatty liver disease (90). For example, a metagenomic analysis of type 2 diabetes found that butyrate-producing bacteria had a reduced proportion of the overall gut flora, whereas butyrate supplementation could treat diabetes by increasing the integrity of the gut barrier (91–93). Moreover, butyrate may reduce diet-induced barrier dysfunction to improve diet-induced obesity (58, 94). In summary, the improvement of metabolic diseases by butyrate mainly depends on the regulation of the intestinal barrier.

Next, we analyzed the specific mechanism by which butyrate regulates the intestinal barrier. Butyrate regulates the intestinal barrier by regulating the physical barrier. In diabetic mice, butyrate stimulates the expression of NLRC3 in colonic epithelial cells, increases the phosphorylation of AMPK, and upregulates tight junction proteins and TJs in colonic epithelial cells. In addition, butyrate can upregulate the transcription of tight junction and mucus genes in epithelial cells (H4 cells), increase claudin-1 expression, and stabilize intestinal epithelial cell functions (60). Additionally, butyrate regulates the repair of the intestinal mucus barrier by activating the macrophage/WNT/ERK signaling pathway (95). Furthermore, butyrate can effectively inhibit the activation, proliferation, and production of cytokines (IFNγ and IL-17) by CD4 T cells, thereby maintaining the intestinal immune barrier and regulating the integrity of the epithelial barrier (96). Numerous studies have also shown, butyrate affects epithelial O2 consumption through epithelial β-oxidation and maintains the stability of hypoxia-inducible factor (HIF). HIF is a transcription factor that coordinates the protection of the barrier, which is essential to maintain the integrity of the intestinal barrier (97–99). Therefore, the treatment and prevention of metabolic diseases from the perspective of butyrate and improving the intestinal barrier are considerable goals, highlighting the need to maintain normal levels of butyrate in the gut during metabolic disease.

Increased levels of intestinal butyrate have been reported in metabolic disease states, notably through the supplementation of butyrate production and the number of bacteria producing butyrate, including *Clostridium butyricum* and *F. prausnitzii*. Other studies have demonstrated that adding modified high-amylose maize-resistant starch increases butyrate concentrations in feces and plasma, thereby decreasing blood sugar levels (31). In addition, the fructooligosaccharide supplement was sufficient to increase butyrate levels, contributing to the balance of host energy. These supplements can be fermented into short-chain fatty acids, such as butyrate, by the hydrolytic enzyme system of beneficial bacteria. Specific genetically modified *Escherichia coli* can promote butyrate levels in the intestine by increasing the production capacity of butyrate (100). A limited number of human studies have shown that butyrate has a good clinical effect in improving the intestinal barrier in metabolic diseases. However, more clinical studies are needed to further confirm its effectiveness and safety in treating metabolic disorders. In addition, the delivery of butyrate to peripheral tissues *via* oral administration is poor because it is absorbed and metabolized by the colon and liver. In treating metabolic diseases, the therapeutic action of butyrate is mainly exerted *via* improvements in the intestinal barrier to enhance the intestinal microenvironment and physiological function, rather than directly affecting peripheral tissues and organs (61, 63). The energy source of colon cells mainly depends on SCFA, especially butyrate, so it may improve the intestinal barrier by improving the function of intestinal epithelial cells. In a study of sodium butyrate for the treatment of intestinal inflammation, sodium butyrate was found to reduce harmful pathogenic bacteria in the gut (such as *Bacteroides, Clostridium, Helicobacter pylori*, and *Desulfovibrio*), suggesting that sodium butyrate may improve the intestinal barrier by beneficially modulating the intestinal microbiota (101, 102).

#### 4.1.3. Berberine

A recent study on berberine demonstrated its metabolic and pathophysiological roles in metabolic disorders, suggesting that it plays a promising role in metabolic diseases, such as obesity, NAFLD, diabetes mellitus, and hyperlipidaemia. For example, it promotes insulin secretion, improves insulin resistance, inhibits adipogenesis, reduces adipose tissue fibrosis, reduces liver steatosis, and improves intestinal flora disturbances (103). Notably, similar studies further confirmed that the function of berberine in the treatment of metabolic diseases largely depends on regulation of the intestinal barrier (64, 104). In addtion, berberine supplementation can improve intestinal flora, regulate innate immunity and improve energy metabolism (105).

The improvement of intestinal barrier function by berberine is multifaceted. First, berberine modulates the intestinal barrier by regulating the physical barrier. The treatment of diabetic rats with berberine restored tight junction protein expression in the intestinal epithelial cells and improved the intestinal barrier (65). In addition, berberine can directly modulate the function of intestinal epithelial cells to repair damaged intestines by promoting differentiation of intestinal stem cells and enhancing cell migration. Second, berberine was found to significantly reduce chronic intestinal inflammation to maintain the intestinal immune barrier function (66). Further studies found that berberine ameliorated pro-inflammatory cytokine-induced tight junction damage in the intestinal epithelium by downregulating the aberrant activation of the TNF-α-NF-κB-MLCK pathway or inhibiting TNF-α, thereby upregulating the expression of tight junction proteins (67). In addition, berberine can ameliorate mucosal inflammation by modulating intestinal epithelial cell and immune cell interactions. Third, by modulating the intestinal barrier through the mucus barrier, oral berberine significantly increased the transcription of mucus-secreting genes and the production of host mucus proteins (68). In addition, berberine also stimulated the growth of the probiotic *Akkermansia*, suggesting that berberine’s improvement of the intestinal barrier may be dependent on the restoration of beneficial intestinal bacterial populations (106).

Notably, although the natural alkaloid berberine has shown promising results in the treatment of the intestinal barrier and metabolic diseases, its clinical application is hampered by its poor gastrointestinal absorption, low bioavailability, and gastrointestinal side effects. In this context, metabolomics and proteomics can be used to explore the mechanism of its treatment of metabolic diseases and study its pharmacokinetics, metabolism, and general safety *in vivo* to improve the intestinal barrier more effectively, while ensuring that it has fewer side effects in humans, thus treating diabetes from multiple perspectives.

#### 4.1.4. Drugs that regulate intestinal inflammation

The pathological state of intestinal inflammation leads to the recruitment of large numbers of immune cells and the release of inflammatory mediators, which affects the physical function of the intestinal barrier by disrupting the structure and intestinal epithelial intercellular junctions (74) and inhibiting intestinal mucosal repair (72). A large body of research has now confirmed that modulating intestinal inflammation is another strategy for restoring and improving impaired intestinal barriers and that intestinal immunomodulatory drugs focus on inhibiting immune factor release and immune cell activation in the first place. In the following, we present an overview of intestinal immunomodulatory drugs, highlighting that they improve intestinal barrier function as evidence and mechanisms for treating metabolic diseases (73).

According to the literature, immunomodulatory drugs can be divided into two categories based on the inhibition of inflammatory factors and the modulation of immune cells (75). First of all, immunomodulatory drugs can reduce the outbreak of intestinal inflammation by decreasing the inflammatory factors in the abnormal outbreak of metabolic diseases and the damage of inflammatory factors to the intestinal barrier and reducing the outbreak of intestinal inflammation (107). It has been noted that the eruption of intestinal pro-inflammatory cytokines (76), such as tumor necrosis factor-a (TNF-a), interleukin (IL)-1, IL-6, IL-9, IL-13, and IL-33, plays a role in the impairment of the intestinal barrier. Anti-IL-6 and anti-TNF-α therapy have been found to improve intestinal permeability by inhibiting the intestinal inflammation caused by the explosion of inflammatory factors (108), possibly mediated through the restoration of expression of intestinal tight junction protein zona occludens-1 (ZO-1) and occludin (109). They are also thought to regulate the reduction of intestinal pro-inflammatory factors, including the iron-binding glycoprotein lactoferrin (LF) and citrin, to restore impaired intestinal barrier function. The close interaction between intestinal epithelial cells and immune cells is essential for maintaining intestinal barrier function (79). Myocardial fibrosis improvement in type 2 diabetes has been reported in commonly used rat models of diabetes, and a recent study implicated it as a receptor-interacting protein kinase 1 (RIPK1) inhibitor (77). RIPK1 inhibitors have been shown to maintain the balance of the immune microenvironment, which normally improves the intestinal barrier by inhibiting the interaction between intestinal epithelial cells (IECs) and immune cells *in vivo* and *in vitro* (110).

Although a large number of studies have shown that systemic inflammation is a potential driver for the development of metabolic diseases, it is believed that interventions to treat inflammation add to the burden of disease and complexity of healthcare. Therefore, we propose that personalized intestinal inflammatory interventions to improve the intestinal barrier for the treatment of metabolic diseases may be a future development in the management of diseases, such as obesity, T2DM, and NAFLD (111). However, the measures required to design effective personalized inflammatory interventions for treatment require considerable refinement. Therefore, in the future, more detailed information will be needed in clinical trials of drugs for the control of metabolic diseases than is currently documented in trials, and is essential for repurposing or developing immunomodulatory therapies to treat metabolic diseases.

### 4.2. Regulating the composition of the intestinal flora to improve the intestinal barrier

**Figure 2:**
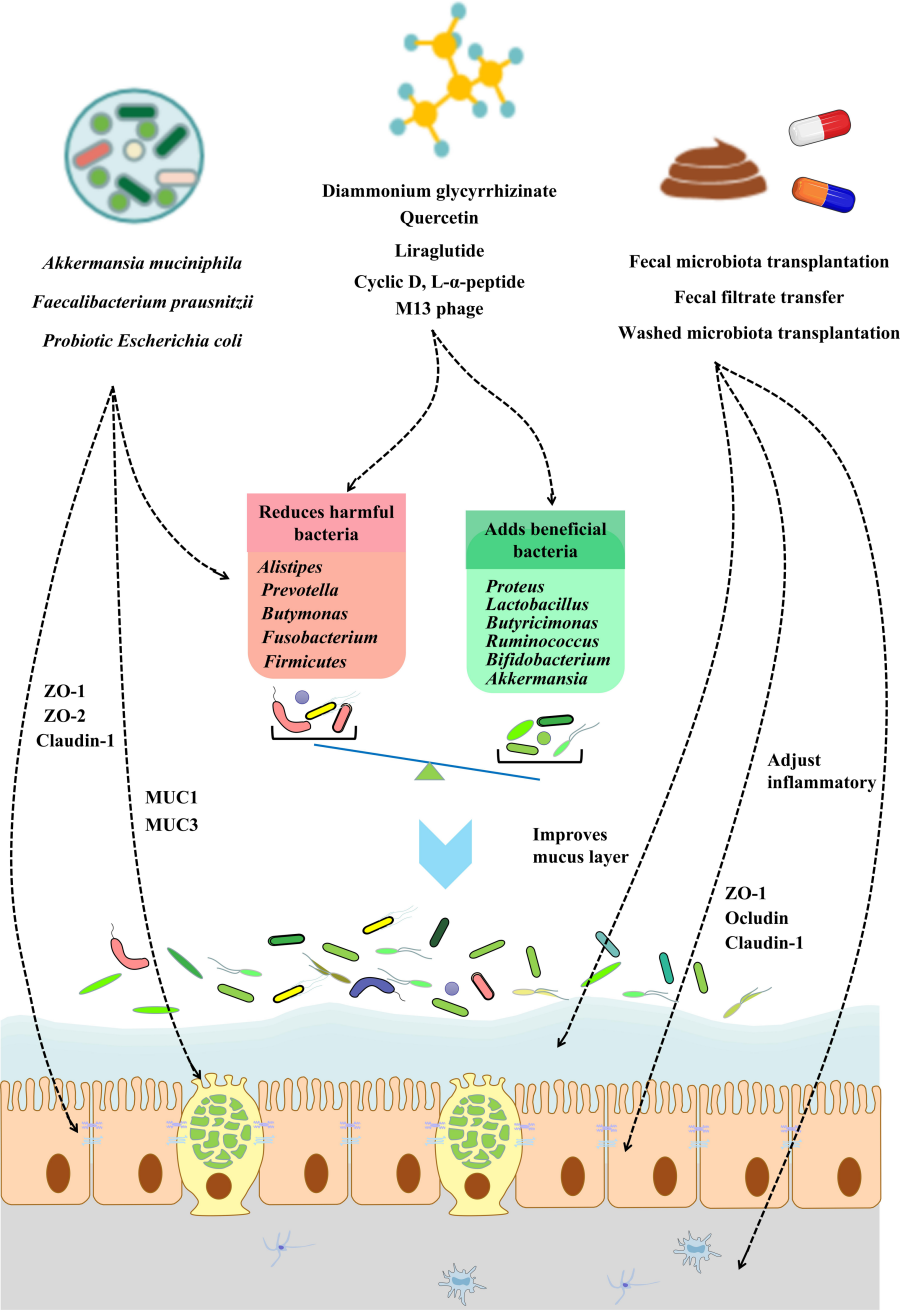
The role of modulating gut microbiota in improving gut barrier integrity. The gut barrier can be affected directly or indirectly by the gut microbiota. The modulation of the gut microbiota can occur *via* probiotics, small-molecule compounds, and fecal transplantation. Restoring the gut microbiota balance can restore gut barrier integrity *via* gut epithelial cell function and tight junction protein expression, improving the mucus barrier and adjusting inflammatory and inflammation. Further improvements of the intestinal barrier can be used to relieve or treat metabolic diseases.

Over the past 20 years, research has shown that the gut flora under normal physiological conditions may help maintain the metabolic health of the human host (5). Further studies have found that metabolic disease states, such as obesity, dyslipidaemia, insulin resistance, and low inflammation, often lead to dysbiosis of the intestinal flora. Intestinal dysbiosis is manifested by an increased abundance of “pro-inflammatory” bacterial strains, such as *Ruminococcus gnavus* or *Bacteroides* species, in the gut, while “anti-inflammatory” strains, such as *Faecalibacterium prausnitzii*, show low abundance (112, 113). Microbial dysbiosis further aggravates the development of metabolic diseases by inducing the disruption of the intestinal barrier (114). A study of T2D patients found that diabetes can cause excessive growth of intestinal flora, increase intestinal permeability, and damage the intestinal barrier (115, 116). The conclusion drawn from extensive data is that dysbiosis of the intestinal flora leads to disruption of the intestinal barrier, mainly through the induction of increased intestinal oxidative stress in the intestinal epithelium, reduced expression of tight junction proteins (such as claudin, occludin, and zonula occludens), and increased mucus degradation (117, 118). Therefore, restoring intestinal barrier function by regulating intestinal flora dysbiosis represents a new approach for the prevention and treatment of metabolic diseases. Currently, there are three ways to modulate the composition of gut microbiota: (i) supplement probiotics, (ii) intervene with specific microbial species using drugs, and (iii) transplant normal intestinal flora to restore the normal intestinal flora ecosystem. The specific mechanism may involve correcting intestinal flora disturbance, increasing the expression of intestinal barrier function proteins, maintaining the normal function of intestinal epithelial cells, and improving intestinal barrier function (119, 120) (Figure 2).

#### 4.2.1. Probiotic supplementation

In the last decade, probiotics [gram-negative anaerobic bacteria *Akkermansia muciniphila* (121), *Lactobacillus reuteri* (122), and gram-positive anaerobic bacteria *Bifidobacterium* (123)*, Roseburia intestinalis* (124)] have been widely used to prevent and treat various diseases, especially metabolic diseases, such as obesity, diabetes, non-alcoholic fatty liver, and hyperlipidaemia, and to improve the microecological balance in the host gut to inhibit the proliferation of harmful bacteria and improve the barrier function of the gastrointestinal tract for the treatment of metabolic diseases (125). In this section, we explore the precise mechanisms by which improving the gut barrier can be used to treat metabolic diseases.

Although probiotics are the most commonly used substances that regulate intestinal flora homeostasis, which can strengthen the intestinal barrier by enhancing epithelial defense function and regulating intestinal microbiota, their application continues to face several challenges (126, 127). Studies have shown that *lactobacillus* maintains intestinal epithelial regeneration and repairs damaged intestinal mucosa (128). Numerous studies have shown that probiotics improve the mechanism of the intestinal barrier by influencing the renewal of intestinal epithelial cells, increasing the production of tight junction proteins, and increasing mucin secretion while promoting the immune system.

The use of probiotic *Bifidobacterium bifidum* strains in obese individuals has shown to improve gut barrier function (129, 130). *Akkermansia muciniphila* and its derived extracellular vesicles (AmEVs) can exert anti-diabetic effects by reducing intestinal barrier disruption and insulin resistance (131, 132). In addition, 14 probiotic species have been found to improve intestinal barrier function in db/db mice (114). Probiotics can improve intestinal epithelial cell function and tight intercellular junctions, and restore intestinal physical barrier function (133). *Escherichia coli* strain Nissle 1917 (EcN) is a Gram-negative probiotic found to modulate the expression and localization of intestinal tight junction proteins, and consequently, enhance intestinal barrier function (134, 135). The probiotic EcN repairs the intestinal epithelial barrier by decreasing the secretion of inflammatory cytokines while increasing the production of anti-inflammatory factors, eliminating reactive oxygen species at the site of inflammation, and effectively relieving symptoms of inflammation (136, 137). Furthermore, EcN *in situ* production of therapeutic protein matrices consisting of coiled nanofibers of trefoil factors (TFFs) can promote mucosal healing and restore mucus barrier function (138–140). In addition, probiotic therapy protects the intestinal epithelial barrier by mitigating a reduction in the expression of tight junction proteins that caused by an intestinal ecological disorder in metabolic diseases and increasing the rate of apoptosis (122, 141). Probiotics are widely used in the treatment of NAFLD as they improve the function of the intestinal immune barrier and inhibit the proliferation and translocation of harmful bacteria. Additional studies have shown that *Bifidobacterium* can enhance the function of the intestinal mucus layer and phagocytes through the activation of intestinal autophagy and calcium signaling pathways (142). Probiotic formulations have also been found to induce an increased expression and secretion of mucin (MUC1 and MUC3) in colonic epithelial cells, improving intestinal mucus barrier function (143). In general, these findings contribute to a better understanding of the complex and beneficial interactions between probiotics and colonic epithelial cells in the gut to restore impaired intestinal barrier function in metabolic diseases (144).

Further studies should screen out effective probiotics *via* the high-throughput analysis of the intestinal flora and, at the same time, explore how probiotics restore the imbalance of the intestinal flora. This is expected to improve the barrier function of the gut by targeting specific probiotics to modulate the dysbiosis of the intestinal flora. With advances in technology, the targeting proteins of probiotics should be further identified through metabolomics and proteomics to provide a reliable theoretical basis for probiotic targeting and to improve the intestinal barrier. Oral probiotics are affected by metabolic disease-induced pathological inflammatory microenvironments, such as reactive oxygen species (ROS) and depleted mucus layers, which limit their survival and their colonization of the gut (137). Therefore, improving probiotic intestinal colonization by modulating the intestinal microenvironment or modifying probiotics is expected to provide an important perspective for the treatment of various metabolic diseases. In a previous study, a synthetic biology approach was used to develop an engineered probiotic with superior resistance to the harsh environment of the gastrointestinal tract to enhance the colonization and growth of probiotics in the mucus layer (145). In addition, through a composite biomagnetic material composed of tiny magnetic particles and probiotics, an external magnetic field can capture and retain probiotics in the gastrointestinal tract of mice, thereby improving the accumulation and stable colonization of probiotics under specific conditions. In summary, in-depth research on probiotics has led to the development of a variety of effective and safe probiotics. In the later stages of research, the focus can be shifted to the transformation of probiotics to make them more suitable for the intestinal environment in the disease state to improve the intestinal barrier more effectively, maintain intestinal homeostasis, and exert their therapeutic effect on metabolic diseases.

#### 4.2.2. Small molecule compounds engineer the gut microbiome

In some ways, the gut microbiota is the largest “organ” of the body, and its composition is species-diverse. Recently, it has been proposed that gut microbiota is involved in the development and progression of metabolic diseases. Host health and disease status can be maintained and improved by modulating the imbalanced gut microbiota, including beneficial and harmful bacteria, based on the symbiotic or antagonistic relationships between various microorganisms. The “gut microbiota barrier axis” can be used as an alternative target for the treatment of metabolic diseases. It restores the impaired intestinal barrier in metabolic disease states by altering the structure and composition of intestinal flora.

Specifically, diammonium glycyrrhizinate (DG) was found to reduce the ratio of *Firmicutes* to *Bacteroidetes* and endotoxin-producing bacteria, such as *Desulfovibrio*, and increase probiotics, such as *Proteus* and *Lactobacillus*, in animal models (146). It also increased the levels of short-chain fatty acid (SCFA)-producing bacteria such as *Ruminococcus* and *Lachnospira* (147), significantly alleviating low-grade intestinal inflammation, improving tight junction protein expression, goblet cell number, and mucin secretion, and enhancing intestinal barrier function to prevent non-alcoholic fatty liver disease in mice (146). In NAFLD treatment studies, obeticholic acid was found to lessen endotoxemia and inflammation levels by reversing gut flora imbalance, particularly increasing the abundance of *Blautia*, and restoring gut barrier function to improve NAFLD (148, 149). Furthermore, the therapeutic effects of liraglutide may be due to improved gut microbiota structure associated with hepatic steatosis (150). In a study on obesity and diabetes, it was found that the intestinal flora can be adjusted by drugs, such as Akebia saponin D (151) and Ganoderma lucidum (152) which significantly reduce the HFD-related *Alistipes* and *Prevotella*, increase the proportion of *Butyricimonas, Ruminococcus*, and *Bifidobacterium*, and increase the abundance of the anti-obesity bacterium *Akkermansia*. Reverse HFD-induced gut dysbiosis, such as reduced *Firmicutes* to *Bacteroidetes* ratios and endotoxin-carrying *Proteobacteria* levels, maintains gut barrier integrity, reduces metabolic endotoxemia, and improves obesity and diabetes (152, 153).

Overall, these results led us to speculate that these drugs could be used to treat metabolic diseases by improving the composition and structure of intestinal microbiota in mice to reduce intestinal permeability. However, the structure of the intestinal flora is complex and rich in diversity. The regulation of intestinal flora by common drugs is often accompanied by interference with the growth of normal intestinal flora.

Next, we discuss the therapeutic potential of targeting gut microbiota based on existing research. Cyclic D-and L-alpha-peptides, using an *in vitro* drug screening protocol, were selected to improve the integrity of the intestinal barrier and inhibit the development of atherosclerosis through the molecular reprogramming of the microbiome transcriptome *via* the selective alteration of bacterial growth. In a colorectal cancer (CRC) study, a specific M13 phage was screened using phage technology to achieve the specific clearance of Fusobacterium nucleatum and to remodel the tumor immune microenvironment (151). Recent studies have demonstrated a correlation between gut phage composition and host health, phage therapy as an antibacterial agent, and the application of genetically engineered phages in gut microbiome remodeling (154, 155). Directed chemical manipulation provides additional tools for deciphering the chemical biology of the gut microbiome and designing phage-containing supplements to target remodeling of the gut microbiota (156). The elimination of specific pathogens can correct gut dysbiosis and improve gut barrier function (157). Therefore, the targeted remodeling of the microbiome should be explored in the future for the treatment of metabolic diseases (158). In a colorectal cancer (CRC) study, a specific M13 phage was screened using phage technology to achieve specific clearance of *Fusobacterium nucleatum* and to remodel the tumor immune microenvironment. Recent studies have demonstrated a correlation between gut phage composition and host health, phage therapy as an antibacterial agent, and the application of genetically engineered phages in gut microbiome remodeling (159, 160). Directed chemical manipulation provides additional tools for deciphering the chemical biology of the gut microbiome and designing phage-containing supplements to target remodeling of the gut microbiota. The elimination of specific pathogens can correct gut dysbiosis and improve the gut barrier. Therefore, the targeted remodeling of the microbiome should be explored in the future for the treatment of metabolic diseases.

#### 4.2.3. Transplanting the normal intestinal flora

Gut microbiota transplantation is where gut microbiota from a healthy donor is transplanted into a patient’s gastrointestinal tract. Previously, this therapy was used to treat gastrointestinal diseases caused by pathogenic microorganisms or opportunistic microbial activities (161). However, a growing number of studies have recently reported on the use of fecal microbiota transplantation for metabolic syndrome, diabetes, and other diseases (162). Population studies have found that fecal microbiota transplantation leads to increased insulin sensitivity in patients with metabolic syndrome and improved glucose metabolism, with the effectiveness of treatment depending on improved gut microbiota and changes in plasma metabolites associated with increased beneficial intestinal metabolites (163, 164). The efficacy of fecal transplants in metabolic diseases is well documented and relies on improvement of the intestinal barrier. Washed microbiota transplantation has been shown to effectively improve compromised gut barrier function, significantly reducing the level of endotoxins and thus reducing the symptoms of gout patients (165). The transplantation of normal fecal microbiota into a mouse model of disease was found to normalize intestinal permeability, thereby significantly reducing metabolic endotoxemia, reversing weight gain, and achieving glucose tolerance (166).

With regard to the apparent restorative effect of intestinal flora transplantation on the intestinal barrier, the underlying mechanisms include restoring dysregulated intestinal flora or acting directly on the host intestine to improve the intestinal barrier. Firstly, flora transplantation improves intestinal tight junctions and increases the expression of intestinal barrier function proteins, including ZO-1, occluding, and claudin-1 (167, 168). Simultaneously, it improves the mucus layer components to protect the function of the mucus barrier. In addition, it significantly modulates the function of intestinal epithelial cells and reduces the loss of villi and epithelial cells by inhibiting epithelial cell apoptosis. Moreover, the beneficial effects of FMT on intestinal barrier function can reduce intestinal inflammation and inhibit inflammatory cell infiltration, thereby reducing the level of systemic inflammation and resulting in a significant reduction in systemic endotoxemia (169, 170). Studies have found that flora transplantation reduces intestinal epithelial cell damage caused by pathogens in the gut and restores the damage to the intestinal barrier caused by the dysbiosis of the intestinal flora. In addition, fecal microbiota transplantation reduced Bacteroidetes and Desulfovibrio, altering the imbalance in the gut microbiota and restoring the richness and diversity of intestinal flora (171). Thus, the intestinal inflammation and intestinal mucosal destruction induced by the dysbiosis of the intestinal flora in metabolic diseases are alleviated.

Although most existing studies show that fecal transplantation has beneficial effects, attention should also be paid to its safety, especially in patients with metabolic diseases that are often accompanied by systemic diseases that decrease immunity. Adverse events have been reported in seven patients who received FMT from fecal donors colonized with Shiga toxin-producing Escherichia coli (STEC) in a clinical study (172). In this context, improved screening and pre-transplant management may reduce adverse events. A preliminary study of five patients with CDI showed that the transfer of sterile filtrate from donor feces (FFT) containing bacterial fragments, proteins, antimicrobial compounds, metabolites, and oligonucleotides/DNA rather than intact microorganisms was sufficient to restore normal bowel habits and eliminate symptoms (173). This finding suggests that bacterial components, metabolites, or phages mediate many of the effects of FMT, and that FFT may be an alternative, especially in immunocompromised patients. In addition, another study proposed for the first time that washed microbiota transplantation (WMT) is safer, more precise, and quality-controllable than manual crude FMT (174). Overall, follow-up studies on fecal transplantation should focus on further strengthening its safety under the premise of ensuring efficacy. Modifying and optimizing the intestinal flora before transplantation is necessary, and it will be beneficial to improve the efficacy of intestinal flora transplantation and reduce the occurrence of adverse events.

### 4.3. Lifestyle interventions

An unreasonable lifestyle is one of the main factors leading to the high incidence of modern metabolic diseases (175, 176). Lifestyle interventions, including physical activity and healthy eating habits, have the aim of controlling weight and reducing the risk factors related to metabolic diseases (177). Studies have reported that a lack of physical activity and unhealthy diet are likely to lead to diabetes and significantly increase the risk of major cardiovascular events (178, 179). This emphasizes the importance of lifestyle changes. No matter the current metabolic state, maintaining a healthy lifestyle can reduce the risk of developing metabolic diseases.

#### 4.3.1. Reasonable exercise

Exercise increases the body’s metabolism and regulates the function of the body’s organs (180), and has been regarded as a treatment prescription for metabolic diseases (181). In a clinical study, sustained moderate exercise increased insulin signaling, decreased lipogenesis and weight loss, and reversed the risk factors for metabolic syndrome (182). A randomized controlled trial found that exercise improved metabolic profile and insulin sensitivity, reduced abdominal fat, and maintained liver fat, blood sugar, and cardiorespiratory fitness in patients with type 2 diabetes (183, 184).

A growing body of research has focused on the use of exercise to treat metabolic diseases by improving the gut barrier. In a six-month exercise training study on 30 T2D patients, long-term exercise was found to reduce gut permeability, improve systemic hypoglycaemia and inflammation, and control diabetes (185). In addition, exercise has been shown to reduce HFD-induced obesity and intestinal barrier damage by modulating lipid metabolism (186) or activating the AMPK/CDX2 signaling pathway (187). This suggests a potential mechanism by which long-term exercise can improve gut barrier integrity (188). Furthermore, the function of exercise in the gut barrier is mainly dependent on the modulation of intestinal epithelial cell function and gut microbiota. For example, exercise can upregulate the expression of claudin-1 and occludin proteins, suggesting that exercise may regulate barrier integrity through tight junctions (189). Moreover, exercise can increase the number of beneficial microbial species, enrich the diversity of microbial communities, promote the development of commensal bacteria, and remodel the gut microbial ecosystem, thus protecting the gut barrier, preventing the dysregulation of the gut-liver axis, and reducing circulating LPS levels, thereby helping to relieve chronic inflammation (190, 191).

Although exercise is widely promoted as a healthy habit in contemporary society, excessive exercise represents a significant health concern. Studies have shown that excessive exercise often leads to impaired intestinal epithelial barrier integrity and gastrointestinal disease. Thus, determining the optimal exercise dose with which to manage metabolic diseases is vital. Recent studies suggest that metabolic disease can be improved significantly by 30 min of moderate-intensity cardio once a week (192). Therefore, an appropriate amount of exercise is suggested, especially in patients with metabolic diseases. Recent clinical trials have found that dietary exercise programs have shown positive effects. Notably, high-intensity interval training (HIIT) with time-restricted eating (TRE) improves cardiometabolic health in at-risk populations (193). In addition, with plant extracts of polyphenols, glycaemic control improves the oxidative capacity of skeletal muscle and intestinal mucosal function (194). Hence, there is an urgent need for more clinical trials on the delivery of rhythm-exercise diet interventions to investigate the long-term effects and feasibility of these interventions over longer durations.

#### 4.3.2. Diet composition adjustment

Unhealthy dietary patterns can lead to hyperinsulinaemia, insulin resistance, dyslipidaemia, low-grade systemic inflammation, and endotoxemia. These pathological processes are closely related to metabolic diseases, such as obesity, type 2 diabetes, hyperlipidaemia, cardiovascular disease, and non-alcoholic fatty liver disease (195). Furthermore, unhealthy dietary patterns promote altered gut function, leading to gut barrier dysfunction, increased permeability, and microbiota dysbiosis (196). Many population-based dietary intervention studies have found that metabolic diseases can be improved by optimizing the dietary structure and components, such as polyphenol-rich diets, increasing dietary fiber, specific vitamin supplements, and energy-restricted diets, which depend on improving intestinal barrier function (197). In the following, we will take a closer look at these diets and intestinal barrier function.

Polyphenols are a well-known class of bioactive compounds that are widely distributed in the plant kingdom and are abundant in plant-based and plant-derived foods. The biological activity of polyphenols has been studied using various *in vitro* and *in vivo* experimental models. These studies have shown their potential to help maintain health and prevent, delay, or reduce the number of chronic diseases (198). The biological functions of polyphenols include antioxidant, anti-inflammatory, and immunomodulatory activities at the intestinal and systemic levels (199). A study of life interventions in elderly subjects found that a diet rich in polyphenols can reduce serum zonulin levels and improve intestinal permeability. Although the precise molecular mechanism is not fully understood, polyphenols can, directly and indirectly, act on different levels of the intestinal barrier by regulating tight junction function, the production of numerous inflammatory cytokines, and the activation of antioxidant genes. This mechanism may improve the intestinal barrier by increasing the expression of tight junction proteins (ZO-1 and occludin) and mucin, and balancing the immune response interaction in the colon (200, 201). In addition, polyphenols undergo extensive alterations in the gut microbiota, thus affecting the gut microbial ecosystem (201, 202). In conclusion, these findings preliminarily reveal the complex relationship between dietary supplementation with polyphenols, intestinal barrier function, and metabolic diseases. The molecular pathways underlying this function using an integrated multi-omics approach (food components, microbiota, gut proteomics, and metabolomics) provide a theoretical basis for future population studies on polyphenol diets (203). In this context, further population studies will be needed to optimize the formulation of personalized polyphenol dietary interventions.

Dietary fiber contains various plant-based compounds that are not fully digested in the human gut, including insoluble fibers, such as cellulose, hemicellulose, and lignin, and soluble fibers, such as pectin, beta-glucan, and hydrocolloids (204). Dietary fiber is a crucial component of the diet. A meta-analysis of randomized controlled trials found that intake of soluble fiber supplements was effective in controlling blood glucose and improving insulin resistance and BMI levels in patients with type 2 diabetes (205). Results of the large-scale NutriNet-Santé prospective cohort study (2009–2019) showed that dietary fiber intake was inversely associated with the risk of mortality from several chronic diseases (cardiovascular disease, cancer, type 2 diabetes) (206). Studies have shown that chronic or intermittent dietary fiber deficiency can lead to the erosion of the colonic mucus barrier and intestinal barrier dysfunction (207, 208). Dietary fiber treatment can increase the thickness of mucus and the number and function of goblet cells (23), and prevent the increase of mucus permeabilityreduce mucus thickness, inflammation, and intestinal damage in mice (209), thereby strengthening the intestinal barrier (210). Specifically, fiber supplements have been found to improve the intestinal barrier of C57BL/6 and Ldlr^−/−^ mice by increasing the colonic mucin layer, reducing systemic inflammation, and significantly reducing WD-induced metabolic disease. In addition, a high-fiber diet can improve intestinal barrier function by regulating immune regulatory cells and increasing intestinal tight junction proteins, thereby reducing the development of autoimmune hepatitis (211). In addition, dietary fiber intervention correlates with host gut microbiota. While identifying beneficial bacterial strains, dietary fiber reshapes the gut microbiome in metabolic diseases according to the preference of bacteria that use the specific and ingested dietary fiber (212). For example, the beneficial effect of dietary fiber on T2D is achieved by increasing butyrate levels and the abundance of beneficial bacteria (*Lachnobacterium, Parabacteroides, Faecalibacterium, Akkermansia*, some butyrate-producing bacteria and SCFA-producing strains) (213), while also reducing 12α-hydroxylation Production of bile acids, acylcarnitines, and metabolically harmful compounds such as indole and hydrogen sulfide (214). Thus, additional well-studied types and sources of dietary fiber are needed to determine the role of metabolic diseases and how dietary fiber diets become precision nutrition and metabolic disease treatment strategies by design.

Vitamin consumption through diet is crucial for controlling a variety of physiological functions, including metabolism. The intestinal tract is the primary absorption site for vitamins A, D, E, and K in the human diet (215).According to research, vitamins A and D may have an impact on the onset of obesity, type 2 diabetes, liver steatosis, and steatohepatitis (216, 217). Additionally, there is growing proof that intestinal barrier function will be harmed by vitamin deficit or excess (218). The topic of enhancing the intestinal barrier with vitamin supplements to treat metabolic illnesses will be covered next (219). First, the study discovered that by increasing tight junction protein expression, vitamins A and D can enhance intestinal barrier function (220–222). Specifically, vitamin A and vitamin D can strengthen the intestinal epithelial barrier function by stabilizing the mucosal immune system, thus affecting the process of intestinal inflammation (223). Additionally, the administration of vitamin A and vitamin D can influence intestinal microflora, including elevating the number of helpful bacteria (like *Clostridaceae*) and lowering the number of pathogenic bacteria (like *Streptococcaceae*) (224), which can also improve the relevant metabolites of the intestinal microflora, like elevating the production of SCFA (225), which can effectively improve metabolic diseases and restore the intestinal barrier function. Recent research has also demonstrated the ability of VB12 oral supplement to participate in the epigenetic modification of intestinal barrier genes, limit the colonization of harmful bacteria, and coordinate the functions of ileal epithelial cells (iEC) and intestinal microbiota (226). Although vitamins have a substantial causal role in metabolic disorders, further research is needed on how specific vitamin intake and type (whether in excess or deficiency) affect the gut barrier, gut bacteria, and the potential to treat metabolic disease.

Investigating the effects of dietary energy restriction (ER) is an active area of research. Dietary ER protocols involve dietary regimens associated with limiting the total daily energy intake or allocating energy intake to specific periods of the day. Dietary ER has been shown to be feasible and effective for weight loss, as well as for the treatment of other metabolic diseases by improving insulin sensitivity and inflammatory markers (227). In addition, one study found that the expression of the tight junction markers claudin-2 and zonula occludens-1 was elevated in the colon of mice in the ER-treated group, suggesting that ER may treat metabolic diseases by regulating the intestinal barrier (228, 229). It should not be overlooked that both energy and protein deficiencies may contribute to age-related bone loss, and patients with osteopenia or osteoporosis must exercise caution when implementing severe energy restrictions (230, 231). In addition, although energy-restricted diets are effective in weight loss, they are difficult to maintain after the resumption of feeding. Studies have found crosstalk between microbiota and bile acids in weight regain and the addition of *Parabacteroides distasonis*, a potential probiotic that could prevent rapid weight gain after calorie restriction diets (232). Further research into dieting and weight regain in humans is therefore necessary to develop nutritional supplements to replace the beneficial bacteria that individuals lose and reduce the incidence of malnutrition.

#### 4.3.3. The spatiotemporal regulation of the diet

High-frequency excessive food intake and an irregular diet often lead to metabolic diseases, intestinal barrier damage and intestinal dysfunction (233). Studies have found that dietary content and rhythm regulate transcription in the intestinal epithelial cells. Changes in the timing or content of feeding can lead to intestinal epithelial cell homeostasis and disrupt intestinal barrier function (234). Multiple trials in adult populations worldwide have examined the efficacy of various dietary timing regimens, including time-restricted eating, short-term fasting, and intermittent fasting. Time-restricted eating involves shortening the eating window to a pre-specified number of hours per day (6 to 10 h) and fasting for several hours the remainder of the time without changing diet quality and quantity (235). Time-restricted eating improves insulin sensitivity, beta-cell responsiveness, blood pressure, oxidative stress, and appetite (236). Clinical studies have found that time-restricted eating reduces the risk of metabolic diseases in healthy individuals when treating metabolic syndrome (237). For instance, time-restricted eating can lower HbA1c levels in individuals with diabetes, which enables achieving blood sugar control and weight loss (238). A previous study found that a 12-week time-restricted eating intervention in 19 subjects with metabolic syndrome improved cardiometabolic health in treating metabolic syndrome (237). Abnormal feeding timing and increased gut permeability are associated with obesity, which in turn modulates feeding rhythms and improves gut barrier function, providing new opportunities to combat metabolic dysfunction (239).

Recent studies have found that dietary rhythms can regulate the bi-directional interaction between the intestinal circadian clock, gut microbiota, and host metabolic system, and enhance the circadian rhythm of adipocytes to improve metabolism (240). In addition, studies on short-term fasting have found that it can protect the viability of small intestinal stem cells in the small intestine of mice and act as a barrier (241, 242). A recent study found that food stimulation activation-induced expression of the neuropeptide vasoactive intestinal peptide significantly enhanced IL-22 production and epithelial barrier function (243). Intermittent eating with moderate rhythm balances the body’s energy intake, regulates intestinal homeostasis, and improves the intestinal barrier through food stimulation and rhythm. In conclusion, regulating feeding timing may improve metabolic diseases through gut barrier function. Although the underlying mechanism has not been fully elucidated, animal experiments have yielded impressive data on the prevention or reversal of obesity-related metabolic diseases. Therefore, more rigorous human studies are needed to assess the efficacy, mechanisms, and sustainability of the meal-timing modulation of the gut barrier in a wide range of populations and diseases. It has been suggested that time-restricted eating may improve the efficacy of pharmacological treatment. Therefore, further population studies should be conduced in the future to explore how time-restricted eating can improve the pathways associated with metabolic diseases.

### 4.4. Metabolic surgery

Bariatric surgery is an effective treatment for patients with metabolic disorders and includes gastric bypass (RYGB) and sleeve gastrectomy (SG), the two most commonly performed procedures (244). These account for 76% of the procedures currently performed in bariatric surgery. They have shown surprising efficacy in improving hyperglycaemia, insulin sensitivity, hyperlipidaemia, and steatosis in patients with metabolic diseases (245). As bariatric surgery remodels the digestive tract, the specific mechanisms for the treatment of metabolic disorders can be further explored in terms of gut function (246–248). A study on SG in morbidly obese patients found that the procedure can significantly improve intestinal barrier damage (249).

Specifically, metabolic surgery improved the intestinal barrier by relying primarily on the increased expression of the intestinal epithelial tight junction proteins ZO-1, occludin, and claudin-1 to maintain intestinal epithelial cell proliferation and restore the physical barrier of the intestine (250). This optimizes mucosal function, increases submucosal thickness, and improves the intestinal mucosal barrier. In addition, it increases the number of Paneth cells and the depth of the crypt, alleviates the intestinal inflammatory response, and enhances the intestinal immune barrier (251). In addition, bariatric surgery can increase the intestinal secretion of molecules, such as glucagon-like peptide 1 (GLP-1) (252) and glucagon-like peptide 2 (GLP-2) (253). Previous studies have shown that the intestinal secretion of GLP-2 inhibits epithelial cell apoptosis and promotes cell proliferation. In addition, GLP-1 can increase intestinal gland secretion and mucin expression, protecting the intestinal barrier (254). Therefore, we speculate that bariatric surgery may treat metabolic diseases by enhancing intestinal barrier function by increasing the secretion of GLP-1 and GLP-2 in the intestine (255). Interestingly, the serum levels of bile acids, such as tauroursodeoxycholic acid (TUDCA) and lithocholic acid (LCA), increase after bariatric surgery (256, 257). LCA and TUDCA can improve intestinal barrier function by reducing intestinal inflammation, suggesting that bariatric surgery improves bile acid metabolism and may further strengthen intestinal barrier function for the treatment of metabolic diseases (258–260).

**Figure 3:**
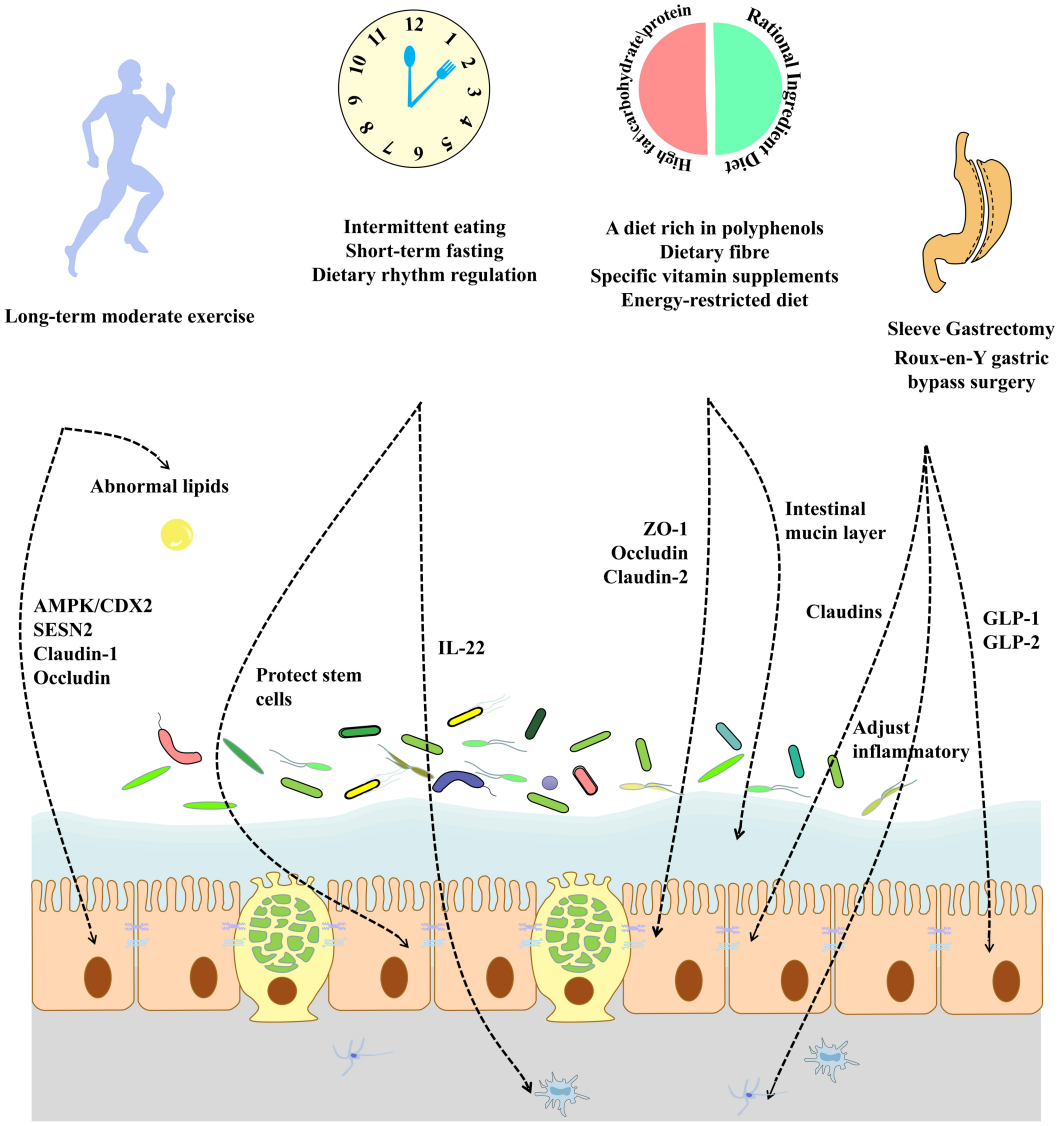
An unhealthy lifestyle and diet can play an important role in the development of metabolic diseases by affecting the intestinal barrier. Exercise over the long-term improves intestinal tight junction protein expression and ameliorates abnormal lipids in the gut. The modulation of dietary rhythms can improve intestinal stem cell function, alleviate chronic inflammation, and improve the intestinal barrier. The modification of the dietary composition increases tight junction protein expression while improving the intestinal mucus barrier. Bariatric surgery in obese patients can adjust intestinal inflammatory and inflammation and increase the secretion function of intestinal epithelial cells by increasing the expression of intestinal tight junction proteins.

Existing research has focused on the management of metabolic diseases through weight loss and energy restriction in bariatric surgery. However, complications, such as protein malnutrition, micronutrient deficiencies, and small intestinal bacterial overgrowth, which can occur after bariatric surgery, should not be overlooked. Therefore, metabolic disorders may be better managed through supplemental pharmacological interventions and the development of new surgical modalities. Further improvements in the intestinal mucosa, enteric nervous system, hormonal responses, and intestinal barrier function after gastric bypass surgery were achieved through supplementation with α-ketoglutarate (261). Compensatory antibody responses may help reduce systemic inflammation by neutralizing the immunogenic components of the intestine, thereby enhancing intestinal barrier function after bariatric surgery. Therefore, the modulation of the postoperative intestinal immune response is a potential strategy. Further studies have shown that endoscopic sleeve gastroplasty is a safer intervention that can result in significant weight loss and reduced postoperative complications. Future research could focus on improving the intestinal barrier to develop a more rational approach to bariatric surgery by restoring intestinal barrier function in patients with metabolic diseases, reducing chronic inflammation both in the gut and systemically, and improving the secretion of hormones, such as GLP-1, in the gut. Serum metabolomics and proteomics should also be used to search for key effectors to reveal the role of gut barrier-targeted bariatric surgery in the treatment of metabolic diseases, such as improved host metabolic disorders, insulin sensitivity, and adipokine secretion, as well as to develop effective postoperative interventional agents to further reduce the incidence of postoperative complications (Figure 3).

## 5. Conclusion

At present, the burden of metabolic diseases, particularly diabetes mellitus, obesity, and NAFLD, is increasing globally. However, there are limited means and efficacy for the treatment of these metabolic diseases. Recent studies have shown that multiple factors in metabolic diseases affect gut barrier function, wherein the impairment of the gut barrier can further exacerbate metabolic disease progression and severity. New therapeutic strategies for manipulating the gut barrier, including drugs, probiotics, diet, or natural products, have been tested clinically and in various diseases, repairing gut barrier dysfunction in many cases. In particular, rational medication coupled with lifestyle interventions represents a safe and effective means to intervene early in chronic metabolic diseases, and may have significant health benefits by modulating the gut barrier. In summary, improving our understanding of the relationship between the gut barrier and metabolic disease can provide detailed mechanistic insights into the pathogenesis and reveal possible pathways for the modulation of disease prevention.

Thus, defining a “healthy” gut barrier and developing new multi-omic technologies in the form of biomarkers and therapeutic tools will significantly advance the research on metabolic diseases. This will allow researchers to investigate the dynamic relationship between metabolic diseases and intestinal barrier function and to provide translational opportunities for therapeutic strategies for metabolic diseases that use the intestinal barrier as a target organ, including drug design, microbial transplantation, and science-based lifestyle. Multi-omics studies can help us to understand the gut barrier-metabolic disease axis and could lead to the development of personalized medicine. Therefore, there is a need to demonstrate causality in metabolic diseases, with a detailed understanding of the gut barrier function, using transcriptomic, proteomic, and metabolomic technologies. The gut barrier-metabolic disease axis is an exciting area of exploration for unraveling the mechanisms that support the therapeutic regulation of metabolic diseases, and an in-depth understanding of these complex systems can be used to develop new preventive and therapeutic strategies.

## Author contributions

YZ, XZ, XY, and PN wrote the manuscript. QG and KY supervised the writing and revised the manuscript. All authors contributed to the article and approved the submitted version.

## Funding

This work was supported by the Natural Science Foundation of Guangxi Zhuang Autonomous Region (2020GXNSFDA297011).

## Conflict of interest

The authors declare that the research was conducted in the absence of any commercial or financial relationships that could be construed as a potential conflict of interest.

## Publisher’s note

All claims expressed in this article are solely those of the authors and do not necessarily represent those of their affiliated organizations, or those of the publisher, the editors and the reviewers. Any product that may be evaluated in this article, or claim that may be made by its manufacturer, is not guaranteed or endorsed by the publisher.

## References

[1] Xu X Yi H Wu J Kuang T Zhang J Li Q. Therapeutic effect of berberine on metabolic diseases: both pharmacological data and clinical evidence. Biomed Pharmacother. (2021) 133:110984. doi: 10.1016/j.biopha.2020.110984, PMID: 33186794

[2] Boutari C Mantzoros CS. A 2022 update on the epidemiology of obesity and a call to action: as its twin COVID-19 pandemic appears to be receding, the obesity and dysmetabolism pandemic continues to rage on. Metabolism. (2022) 133:155217. doi: 10.1016/j.metabol.2022.155217, PMID: 35584732 PMC9107388

[3] Sun H Saeedi P Karuranga S Pinkepank M Ogurtsova K Duncan BB. IDF diabetes atlas: global, regional and country-level diabetes prevalence estimates for 2021 and projections for 2045. Diabetes Res Clin Pract. (2022) 183:109119. doi: 10.1016/j.diabres.2021.109119, PMID: 34879977 PMC11057359

[4] Ye Q Zou B Yeo YH Li J Huang DQ Wu Y. Global prevalence, incidence, and outcomes of non-obese or lean non-alcoholic fatty liver disease: a systematic review and meta-analysis. Lancet Gastroenterol Hepatol. (2020) 5:739–52. doi: 10.1016/S2468-1253(20)30077-7, PMID: 32413340

[5] Fan Y Pedersen O. Gut microbiota in human metabolic health and disease. Nat Rev Microbiol. (2021) 19:55–71. doi: 10.1038/s41579-020-0433-9 32887946

[6] Sayoc-Becerra A Krishnan M Fan S Jimenez J Hernandez R Gibson K. The JAK-inhibitor Tofacitinib rescues human intestinal epithelial cells and Colonoids from cytokine-induced barrier dysfunction. Inflamm Bowel Dis. (2020) 26:407–22. doi: 10.1093/ibd/izz266, PMID: 31751457 PMC7012302

[7] Tyszka M Bilinski J Basak GW. Advances in intestinal barrier preservation and restoration in the allogeneic hematopoietic cell transplantation setting. J Clin Med. (2021) 10:2508. doi: 10.3390/jcm10112508, PMID: 34204044 PMC8201017

[8] Massier L Bluher M Kovacs P Chakaroun RM. Impaired intestinal barrier and tissue bacteria: Pathomechanisms for metabolic diseases. Front Endocrinol. (2021) 12:616506. doi: 10.3389/fendo.2021.616506, PMID: 33767669 PMC7985551

[9] Mao JW Tang HY Zhao T Tan XY Bi J Wang BY. Intestinal mucosal barrier dysfunction participates in the progress of nonalcoholic fatty liver disease. Int J Clin Exp Pathol. (2015) 8:3648–58. PMID: 26097546 PMC4466933

[10] Cani PD Amar J Iglesias MA Poggi M Knauf C Bastelica D. Metabolic endotoxemia initiates obesity and insulin resistance. Diabetes. (2007) 56:1761–72. doi: 10.2337/db06-1491, PMID: 17456850

[11] Chopyk DM Grakoui A. Contribution of the intestinal microbiome and gut barrier to hepatic disorders. Gastroenterology. (2020) 159:849–63. doi: 10.1053/j.gastro.2020.04.077, PMID: 32569766 PMC7502510

[12] Taleb S. Tryptophan dietary impacts gut barrier and metabolic diseases. Front Immunol. (2019) 10:2113. doi: 10.3389/fimmu.2019.02113, PMID: 31552046 PMC6746884

[13] Kessoku T Kobayashi T Tanaka K Yamamoto A Takahashi K Iwaki M. The role of leaky gut in nonalcoholic fatty liver disease: a novel therapeutic target. Int J Mol Sci. (2021) 22:8161. doi: 10.3390/ijms22158161, PMID: 34360923 PMC8347478

[14] Chen R Wu P Cai Z Fang Y Zhou H Lasanajak Y. Puerariae lobatae radix with chuanxiong Rhizoma for treatment of cerebral ischemic stroke by remodeling gut microbiota to regulate +the brain-gut barriers. J Nutr Biochem. (2019) 65:101–14. doi: 10.1016/j.jnutbio.2018.12.004, PMID: 30710886

[15] Nalle SC Zuo L Ong M Singh G Worthylake AM Choi W. Graft-versus-host disease propagation depends on increased intestinal epithelial tight junction permeability. J Clin Invest. (2019) 129:902–14. doi: 10.1172/JCI98554, PMID: 30667372 PMC6355225

[16] Vecchio AJ Stroud RM. Claudin-9 structures reveal mechanism for toxin-induced gut barrier breakdown. Proc Natl Acad Sci U S A. (2019) 116:17817–24. doi: 10.1073/pnas.1908929116, PMID: 31434788 PMC6731655

[17] Laudisi F Stolfi C Bevivino G Maresca C Franze E Troncone E. GATA6 deficiency leads to epithelial barrier dysfunction and enhances susceptibility to gut inflammation. J Crohns Colitis. (2022) 16:301–11. doi: 10.1093/ecco-jcc/jjab145, PMID: 34374415

[18] Yan H Ajuwon KM. Butyrate modifies intestinal barrier function in IPEC-J2 cells through a selective upregulation of tight junction proteins and activation of the Akt signaling pathway. PLoS One. (2017) 12:e0179586. doi: 10.1371/journal.pone.0179586, PMID: 28654658 PMC5487041

[19] Tajik N Frech M Schulz O Schalter F Lucas S Azizov V. Targeting zonulin and intestinal epithelial barrier function to prevent onset of arthritis. Nat Commun. (2020) 11:1995. doi: 10.1038/s41467-020-15831-7, PMID: 32332732 PMC7181728

[20] Li J Zhang L Wu T Li Y Zhou X Ruan Z. Indole-3-propionic acid improved the intestinal barrier by enhancing epithelial barrier and mucus barrier. J Agric Food Chem. (2021) 69:1487–95. doi: 10.1021/acs.jafc.0c05205, PMID: 33356219

[21] Johansson ME Phillipson M Petersson J Velcich A Holm L Hansson GC. The inner of the two Muc2 mucin-dependent mucus layers in colon is devoid of bacteria. Proc Natl Acad Sci U S A. (2008) 105:15064–9. doi: 10.1073/pnas.0803124105, PMID: 18806221 PMC2567493

[22] Alemao CA Budden KF Gomez HM Rehman SF Marshall JE Shukla SD. Impact of diet and the bacterial microbiome on the mucous barrier and immune disorders. Allergy. (2021) 76:714–34. doi: 10.1111/all.14548, PMID: 32762040

[23] Suriano F Nystrom EEL Sergi D Gustafsson JK. Diet, microbiota, and the mucus layer: the guardians of our health. Front Immunol. (2022) 13:953196. doi: 10.3389/fimmu.2022.953196, PMID: 36177011 PMC9513540

[24] Paone P Cani PD. Mucus barrier, mucins and gut microbiota: the expected slimy partners? Gut. (2020) 69:2232–43. doi: 10.1136/gutjnl-2020-322260, PMID: 32917747 PMC7677487

[25] van der Post S Jabbar KS Birchenough G Arike L Akhtar N Sjovall H. Structural weakening of the colonic mucus barrier is an early event in ulcerative colitis pathogenesis. Gut. (2019) 68:2142–51. doi: 10.1136/gutjnl-2018-317571, PMID: 30914450 PMC6872445

[26] Olivares-Villagomez D Van Kaer L. Intestinal intraepithelial lymphocytes: sentinels of the mucosal barrier. Trends Immunol. (2018) 39:264–75. doi: 10.1016/j.it.2017.11.003, PMID: 29221933 PMC8056148

[27] Sun T Nguyen A Gommerman JL. Dendritic cell subsets in intestinal immunity and inflammation. J Immunol. (2020) 204:1075–83. doi: 10.4049/jimmunol.1900710 32071090

[28] Gill PA Inniss S Kumagai T Rahman FZ Smith AM. The role of diet and gut microbiota in regulating gastrointestinal and inflammatory disease. Front Immunol. (2022) 13:866059. doi: 10.3389/fimmu.2022.866059, PMID: 35450067 PMC9016115

[29] Riedel S Pheiffer C Johnson R Louw J Muller CJF. Intestinal barrier function and immune homeostasis are missing links in obesity and type 2 diabetes development. Front Endocrinol. (2022) 12:833544. doi: 10.3389/fendo.2021.833544 PMC8821109 35145486

[30] Tang WHW Li DY Hazen SL. Dietary metabolism, the gut microbiome, and heart failure. Nat Rev Cardiol. (2019) 16:137–54. doi: 10.1038/s41569-018-0108-7, PMID: 30410105 PMC6377322

[31] Mouries J Brescia P Silvestri A Spadoni I Sorribas M Wiest R. Microbiota-driven gut vascular barrier disruption is a prerequisite for non-alcoholic steatohepatitis development. J Hepatol. (2019) 71:1216–28. doi: 10.1016/j.jhep.2019.08.005, PMID: 31419514 PMC6880766

[32] Genser L Aguanno D Soula HA Dong L Trystram L Assmann K. Increased jejunal permeability in human obesity is revealed by a lipid challenge and is linked to inflammation and type 2 diabetes. J Pathol. (2018) 246:217–30. doi: 10.1002/path.5134, PMID: 29984492

[33] Guo Y Li H Liu Z Li C Chen Y Jiang C. Impaired intestinal barrier function in a mouse model of hyperuricemia. Mol Med Rep. (2019) 20:3292–300. doi: 10.3892/mmr.2019.10586, PMID: 31432190 PMC6755192

[34] Kim S Goel R Kumar A Qi Y Lobaton G Hosaka K. Imbalance of gut microbiome and intestinal epithelial barrier dysfunction in patients with high blood pressure. Clin Sci. (2018) 132:701–18. doi: 10.1042/CS20180087, PMID: 29507058 PMC5955695

[35] Li LJ Gong C Zhao MH Feng BS. Role of interleukin-22 in inflammatory bowel disease. World J Gastroenterol. (2014) 20:18177–88. doi: 10.3748/wjg.v20.i48.18177, PMID: 25561785 PMC4277955

[36] Juge N. Relationship between mucosa-associated gut microbiota and human diseases. Biochem Soc Trans. (2022) 50:1225–36. doi: 10.1042/BST20201201, PMID: 36214382 PMC9704521

[37] Schroeder BO Birchenough GMH Pradhan M Nystrom EEL Henricsson M Hansson GC. Obesity-associated microbiota contributes to mucus layer defects in genetically obese mice. J Biol Chem. (2020) 295:15712–26. doi: 10.1074/jbc.RA120.015771, PMID: 32900852 PMC7667970

[38] McPhee JB Schertzer JD. Immunometabolism of obesity and diabetes: microbiota link compartmentalized immunity in the gut to metabolic tissue inflammation. Clin Sci. (2015) 129:1083–96. doi: 10.1042/CS20150431, PMID: 26464517

[39] Stolfi C Maresca C Monteleone G Laudisi F. Implication of intestinal barrier dysfunction in gut dysbiosis and diseases. Biomedicine. (2022) 10:289. doi: 10.3390/biomedicines10020289, PMID: 35203499 PMC8869546

[40] Mokkala K Pellonpera O Roytio H Pussinen P Ronnemaa T Laitinen K. Increased intestinal permeability, measured by serum zonulin, is associated with metabolic risk markers in overweight pregnant women. Metabolism. (2017) 69:43–50. doi: 10.1016/j.metabol.2016.12.015, PMID: 28285651

[41] Natividad JM Lamas B Pham HP Michel ML Rainteau D Bridonneau C. Bilophila wadsworthia aggravates high fat diet induced metabolic dysfunctions in mice. Nat Commun. (2018) 9:2802. doi: 10.1038/s41467-018-05249-7, PMID: 30022049 PMC6052103

[42] Xie Y Ding F Di W Lv Y Xia F Sheng Y. Impact of a high-fat diet on intestinal stem cells and epithelial barrier function in middle-aged female mice. Mol Med Rep. (2020) 21:1133–44. doi: 10.3892/mmr.2020.10932, PMID: 32016468 PMC7003032

[43] Janczy A Aleksandrowicz-Wrona E Kochan Z Malgorzewicz S. Impact of diet and synbiotics on selected gut bacteria and intestinal permeability in individuals with excess body weight - A prospective, randomized study. Acta Biochim Pol. (2020) 67:571–8. doi: 10.18388/abp.2020_5443, PMID: 33326198

[44] Zhou D Pan Q Xin FZ Zhang RN He CX Chen GY. Sodium butyrate attenuates high-fat diet-induced steatohepatitis in mice by improving gut microbiota and gastrointestinal barrier. World J Gastroenterol. (2017) 23:60–75. doi: 10.3748/wjg.v23.i1.60, PMID: 28104981 PMC5221287

[45] Salden BN Troost FJ Wilms E Truchado P Vilchez-Vargas R Pieper DH. Reinforcement of intestinal epithelial barrier by arabinoxylans in overweight and obese subjects: a randomized controlled trial: arabinoxylans in gut barrier. Clin Nutr. (2018) 37:471–80. doi: 10.1016/j.clnu.2017.01.024, PMID: 28214040

[46] Arakawa K Ishigami T Nakai-Sugiyama M Chen L Doi H Kino T. Lubiprostone as a potential therapeutic agent to improve intestinal permeability and prevent the development of atherosclerosis in apolipoprotein E-deficient mice. PLoS One. (2019) 14:e0218096. doi: 10.1371/journal.pone.0218096, PMID: 31206525 PMC6576757

[47] Camara-Lemarroy CR Metz L Meddings JB Sharkey KA Wee YV. The intestinal barrier in multiple sclerosis: implications for pathophysiology and therapeutics. Brain. (2018) 141:1900–16. doi: 10.1093/brain/awy131, PMID: 29860380 PMC6022557

[48] Lewis CV Taylor WR. Intestinal barrier dysfunction as a therapeutic target for cardiovascular disease. Am J Physiol Heart Circ Physiol. (2020) 319:H1227–33. doi: 10.1152/ajpheart.00612.2020, PMID: 32986965 PMC7792706

[49] Wu W Wang S Liu Q Shan T Wang Y. Metformin protects against LPS-induced intestinal barrier dysfunction by activating AMPK pathway. Mol Pharm. (2018) 15:3272–84. doi: 10.1021/acs.molpharmaceut.8b00332, PMID: 29969038

[50] Deng J Zeng L Lai X Li J Liu L Lin Q. Metformin protects against intestinal barrier dysfunction via AMPKalpha1-dependent inhibition of JNK signalling activation. J Cell Mol Med. (2018) 22:546–57. doi: 10.1111/jcmm.13342, PMID: 29148173 PMC5742676

[51] Zhou ZY Ren LW Zhan P Yang HY Chai DD Yu ZW. Metformin exerts glucose-lowering action in high-fat fed mice via attenuating endotoxemia and enhancing insulin signaling. Acta Pharmacol Sin. (2016) 37:1063–75. doi: 10.1038/aps.2016.21, PMID: 27180982 PMC4973377

[52] Zhou HY Zhu H Yao XM Qian JP Yang J Pan XD. Metformin regulates tight junction of intestinal epithelial cells via MLCK-MLC signaling pathway. Eur Rev Med Pharmacol Sci. (2017) 21:5239–46. doi: 10.26355/eurrev_201711_13847, PMID: 29228440

[53] Ahmadi S Razazan A Nagpal R Jain S Wang B Mishra SP. Metformin reduces aging-related leaky gut and improves cognitive function by beneficially modulating gut microbiome/goblet cell/mucin axis. J Gerontol A Biol Sci Med Sci. (2020) 75:e9–e21. doi: 10.1093/gerona/glaa056, PMID: 32129462 PMC7302182

[54] Brandt A Hernandez-Arriaga A Kehm R Sanchez V Jin CJ Nier A. Metformin attenuates the onset of non-alcoholic fatty liver disease and affects intestinal microbiota and barrier in small intestine. Sci Rep. (2019) 9:6668:6668. doi: 10.1038/s41598-019-43228-0, PMID: 31040374 PMC6491483

[55] Wang J Chen C Ren Y Zhou X Yu S. Metformin alleviates intestinal epithelial barrier damage by inhibiting endoplasmic reticulum stress-induced cell apoptosis in colitis cell model. Zhejiang Da Xue Xue Bao Yi Xue Ban. (2021) 50:627–32. doi: 10.3724/zdxbyxb-2021-0242, PMID: 34986539 PMC8732256

[56] Li Y Yang T Yao Q Li S Fang E Li Y. Metformin prevents colonic barrier dysfunction by inhibiting mast cell activation in maternal separation-induced IBS-like rats. Neurogastroenterol Motil. (2019) 31:e13556. doi: 10.1111/nmo.13556, PMID: 30740845

[57] Wang HB Wang PY Wang X Wan YL Liu YC. Butyrate enhances intestinal epithelial barrier function via up-regulation of tight junction protein Claudin-1 transcription. Dig Dis Sci. (2012) 57:3126–35. doi: 10.1007/s10620-012-2259-4, PMID: 22684624

[58] Xu YH Gao CL Guo HL Zhang WQ Huang W Tang SS. Sodium butyrate supplementation ameliorates diabetic inflammation in db/db mice. J Endocrinol. (2018) 238:231–44. doi: 10.1530/JOE-18-0137, PMID: 29941502

[59] Yang T Yang H Heng C Wang H Chen S Hu Y. Amelioration of non-alcoholic fatty liver disease by sodium butyrate is linked to the modulation of intestinal tight junctions in db/db mice. Food Funct. (2020) 11:10675–89. doi: 10.1039/D0FO01954B, PMID: 33216087

[60] Cheng D Xu JH Li JY Wang SY Wu TF Chen QK. Butyrate ameliorated-NLRC3 protects the intestinal barrier in a GPR43-dependent manner. Exp Cell Res. (2018) 368:101–10. doi: 10.1016/j.yexcr.2018.04.018, PMID: 29689277

[61] Gao Y Davis B Zhu W Zheng N Meng D Walker WA. Short-chain fatty acid butyrate, a breast milk metabolite, enhances immature intestinal barrier function genes in response to inflammation in vitro and in vivo. Am J Physiol Gastrointest Liver Physiol. (2021) 320:G521–30. doi: 10.1152/ajpgi.00279.2020, PMID: 33085904 PMC8238162

[62] Chen G Ran X Li B Li Y He D Huang B. Sodium butyrate inhibits inflammation and maintains epithelium barrier integrity in a TNBS-induced inflammatory bowel disease mice model. EBioMedicine. (2018) 30:317–25. doi: 10.1016/j.ebiom.2018.03.030, PMID: 29627390 PMC5952406

[63] Gonzalez A Krieg R Massey HD Carl D Ghosh S Gehr TWB. Sodium butyrate ameliorates insulin resistance and renal failure in CKD rats by modulating intestinal permeability and mucin expression. Nephrol Dial Transplant. (2019) 34:783–94. doi: 10.1093/ndt/gfy238, PMID: 30085297 PMC6503301

[64] Gong J Hu M Huang Z Fang K Wang D Chen Q. Berberine attenuates intestinal mucosal barrier dysfunction in type 2 diabetic rats. Front Pharmacol. (2017) 8:42. doi: 10.3389/fphar.2017.00042 28217099 PMC5290458

[65] Shan CY Yang JH Kong Y Wang XY Zheng MY Xu YG. Alteration of the intestinal barrier and GLP2 secretion in Berberine-treated type 2 diabetic rats. J Endocrinol. (2013) 218:255–62. doi: 10.1530/JOE-13-0184, PMID: 23757509

[66] Li N Gu L Qu L Gong J Li Q Zhu W. Berberine attenuates pro-inflammatory cytokine-induced tight junction disruption in an in vitro model of intestinal epithelial cells. Eur J Pharm Sci. (2010) 40:1–8. doi: 10.1016/j.ejps.2010.02.001, PMID: 20149867

[67] Amasheh M Fromm A Krug SM Amasheh S Andres S Zeitz M. TNFalpha-induced and berberine-antagonized tight junction barrier impairment via tyrosine kinase, Akt and NFkappaB signaling. J Cell Sci. (2010) 123:4145–55. doi: 10.1242/jcs.070896, PMID: 21062898

[68] Zhang D Jiang L Wang M Jin M Zhang X Liu D. Berberine inhibits intestinal epithelial barrier dysfunction in colon caused by peritoneal dialysis fluid by improving cell migration. J Ethnopharmacol. (2021) 264:113206:113206. doi: 10.1016/j.jep.2020.113206, PMID: 32750460

[69] Li H Fan C Lu H Feng C He P Yang X. Protective role of berberine on ulcerative colitis through modulating enteric glial cells-intestinal epithelial cells-immune cells interactions. Acta Pharm Sin B. (2020) 10:447–61. doi: 10.1016/j.apsb.2019.08.006, PMID: 32140391 PMC7049614

[70] Li H Feng C Fan C Yang Y Yang X Lu H. Intervention of oncostatin M-driven mucosal inflammation by berberine exerts therapeutic property in chronic ulcerative colitis. Cell Death Dis. (2020) 11:271:271. doi: 10.1038/s41419-020-2470-8, PMID: 32332711 PMC7181765

[71] Zhang LC Wang Y Tong LC Sun S Liu WY Zhang S. Berberine alleviates dextran sodium sulfate-induced colitis by improving intestinal barrier function and reducing inflammation and oxidative stress. Exp Ther Med. (2017) 13:3374–82. doi: 10.3892/etm.2017.4402, PMID: 28587416 PMC5450762

[72] Noth R Stuber E Hasler R Nikolaus S Kuhbacher T Hampe J. Anti-TNF-alpha antibodies improve intestinal barrier function in Crohn’s disease. J Crohns Colitis. (2012) 6:464–9. doi: 10.1016/j.crohns.2011.10.004, PMID: 22398062

[73] Zeissig S Bojarski C Buergel N Mankertz J Zeitz M Fromm M. Downregulation of epithelial apoptosis and barrier repair in active Crohn’s disease by tumour necrosis factor alpha antibody treatment. Gut. (2004) 53:1295–302. doi: 10.1136/gut.2003.036632, PMID: 15306588 PMC1774168

[74] Guo Y Zhou G He C Yang W He Z Liu Z. Serum levels of lipopolysaccharide and 1,3-beta-D-glucan refer to the severity in patients with Crohn’s disease. Mediat Inflamm. (2015) 2015:1–9. doi: 10.1155/2015/843089 PMC4464677 26106258

[75] Fang L Pang Z Shu W Wu W Sun M Cong Y. Anti-TNF therapy induces CD4+ T-cell production of IL-22 and promotes epithelial repairs in patients with Crohn’s disease. Inflamm Bowel Dis. (2018) 24:1733–44. doi: 10.1093/ibd/izy126, PMID: 29718341

[76] Chi JH Seo GS Lee SH. Oregonin inhibits inflammation and protects against barrier disruption in intestinal epithelial cells. Int Immunopharmacol. (2018) 59:134–40. doi: 10.1016/j.intimp.2018.04.006, PMID: 29655054

[77] Lu H Li H Fan C Qi Q Yan Y Wu Y. RIPK1 inhibitor ameliorates colitis by directly maintaining intestinal barrier homeostasis and regulating following IECs-immuno crosstalk. Biochem Pharmacol. (2020) 172:113751:113751. doi: 10.1016/j.bcp.2019.113751, PMID: 31837309

[78] Liu L Liang L Yang C Zhou Y Chen Y. Extracellular vesicles of *Fusobacterium nucleatum* compromise intestinal barrier through targeting RIPK1-mediated cell death pathway. Gut Microbes. (2021) 13:1–20. doi: 10.1080/19490976.2021.1902718, PMID: 33769187 PMC8007154

[79] Xiong Y Chen D Yu C Lv B Peng J Wang J. Citrus nobiletin ameliorates experimental colitis by reducing inflammation and restoring impaired intestinal barrier function. Mol Nutr Food Res. (2015) 59:829–42. doi: 10.1002/mnfr.201400614, PMID: 25655748

[80] Spalinger MR Sayoc-Becerra A Ordookhanian C Canale V Santos AN King SJ. The JAK inhibitor Tofacitinib rescues intestinal barrier defects caused by disrupted epithelial-macrophage interactions. J Crohns Colitis. (2021) 15:471–84. doi: 10.1093/ecco-jcc/jjaa182, PMID: 32909045 PMC7944512

[81] Saisho Y. Metformin and inflammation: its potential beyond glucose-lowering effect. Endocr Metab Immune Disord Drug Targets. (2015) 15:196–205. doi: 10.2174/1871530315666150316124019, PMID: 25772174

[82] Sansome DJ Xie C Veedfald S Horowitz M Rayner CK Wu T. Mechanism of glucose-lowering by metformin in type 2 diabetes: role of bile acids. Diabetes Obes Metab. (2020) 22:141–8. doi: 10.1111/dom.13869, PMID: 31468642

[83] Sun EW Martin AM Wattchow DA de Fontgalland D Rabbitt P Hollington P. Metformin triggers PYY secretion in human gut mucosa. J Clin Endocrinol Metab. (2019) 104:2668–74. doi: 10.1210/jc.2018-02460, PMID: 30759215

[84] Simbrunner B Trauner M Reiberger T. Review article: therapeutic aspects of bile acid signalling in the gut-liver axis. Aliment Pharmacol Ther. (2021) 54:1243–62. doi: 10.1111/apt.16602, PMID: 34555862 PMC9290708

[85] Faradonbeh FA Sa II Lastuvkova H Cermanova J Hroch M Faistova H. Metformin impairs bile acid homeostasis in ethinylestradiol-induced cholestasis in mice. Chem Biol Interact. (2021) 345:109525:109525. doi: 10.1016/j.cbi.2021.109525 34058177

[86] Reiner J Thiery J Held J Berlin P Skarbaliene J Vollmar B. The dual GLP-1 and GLP-2 receptor agonist dapiglutide promotes barrier function in murine short bowel. Ann N Y Acad Sci. (2022) 1514:132–41. doi: 10.1111/nyas.14791, PMID: 35580981

[87] Rubio C Puerto M Garcia-Rodriquez JJ Lu VB Garcia-Martinez I Alen R. Impact of global PTP1B deficiency on the gut barrier permeability during NASH in mice. Mol Metab. (2020) 35:100954. doi: 10.1016/j.molmet.2020.01.018, PMID: 32244182 PMC7082558

[88] Zheng J Xiao KL Chen L Wu C Hu X Zeng T. Insulin sensitizers improve the GLP-1 secretion and the amount of intestinal L cells on high-fat-diet-induced catch-up growth. Nutrition. (2017) 39-40:82–91. doi: 10.1016/j.nut.2017.01.002, PMID: 28606576

[89] Liu H Wang J He T Becker S Zhang G Li D. Butyrate: a double-edged sword for health? Adv Nutr. (2018) 9:21–9. doi: 10.1093/advances/nmx009, PMID: 29438462 PMC6333934

[90] Matheus VA Monteiro L Oliveira RB Maschio DA Collares-Buzato CB. Butyrate reduces high-fat diet-induced metabolic alterations, hepatic steatosis and pancreatic beta cell and intestinal barrier dysfunctions in prediabetic mice. Exp Biol Med. (2017) 242:1214–26. doi: 10.1177/1535370217708188, PMID: 28504618 PMC5476343

[91] Qin J Li Y Cai Z Li S Zhu J Zhang F. A metagenome-wide association study of gut microbiota in type 2 diabetes. Nature. (2012) 490:55–60. doi: 10.1038/nature11450, PMID: 23023125

[92] Bridgeman SC Northrop W Melton PE Ellison GC Newsholme P Mamotte CDS. Butyrate generated by gut microbiota and its therapeutic role in metabolic syndrome. Pharmacol Res. (2020) 160:105174. doi: 10.1016/j.phrs.2020.105174, PMID: 32860943

[93] Prins GH Rios-Morales M Gerding A Reijngoud DJ Olinga P Bakker BM. The effects of butyrate on induced metabolic-associated fatty liver disease in precision-cut liver slices. Nutrients. (2021) 13:4203. doi: 10.3390/nu13124203, PMID: 34959755 PMC8703944

[94] Beisner J Filipe Rosa L Kaden-Volynets V Stolzer I Gunther C Bischoff SC. Prebiotic inulin and sodium butyrate attenuate obesity-induced intestinal barrier dysfunction by induction of antimicrobial peptides. Front Immunol. (2021) 12:678360. doi: 10.3389/fimmu.2021.678360, PMID: 34177920 PMC8226265

[95] Liang L Liu L Zhou W Yang C Mai G Li H. Gut microbiota-derived butyrate regulates gut mucus barrier repair by activating the macrophage/WNT/ERK signaling pathway. Clin Sci. (2022) 136:291–307. doi: 10.1042/CS20210778, PMID: 35194640

[96] Kibbie JJ Dillon SM Thompson TA Purba CM McCarter MD Wilson CC. Butyrate directly decreases human gut lamina propria CD4 T cell function through histone deacetylase (HDAC) inhibition and GPR43 signaling. Immunobiology. (2021) 226:152126. doi: 10.1016/j.imbio.2021.152126, PMID: 34365090 PMC8478853

[97] Kelly CJ Zheng L Campbell EL Saeedi B Scholz CC Bayless AJ. Crosstalk between microbiota-derived short-chain fatty acids and intestinal epithelial HIF augments tissue barrier function. Cell Host Microbe. (2015) 17:662–71. doi: 10.1016/j.chom.2015.03.005, PMID: 25865369 PMC4433427

[98] Salvi PS Cowles RA. Butyrate and the intestinal epithelium: modulation of proliferation and inflammation in homeostasis and disease. Cells. (2021) 10:1775. doi: 10.3390/cells10071775, PMID: 34359944 PMC8304699

[99] Yin J Zhou C Yang K Ren Y Qiu Y Xu P. Mutual regulation between butyrate and hypoxia-inducible factor-1alpha in epithelial cell promotes expression of tight junction proteins. Cell Biol Int. (2020) 44:1405–14. doi: 10.1002/cbin.11336, PMID: 32129567

[100] Park YT Kim T Ham J Choi J Lee HS Yeon YJ. Physiological activity of *E. coli* engineered to produce butyric acid. Microb Biotechnol. (2022) 15:832–43. doi: 10.1111/1751-7915.13795, PMID: 33729711 PMC8913873

[101] Ma X Zhou Z Zhang X Fan M Hong Y Feng Y. Sodium butyrate modulates gut microbiota and immune response in colorectal cancer liver metastatic mice. Cell Biol Toxicol. (2020) 36:509–15. doi: 10.1007/s10565-020-09518-4, PMID: 32172331

[102] Dou X Ma Z Yan D Gao N Li Z Li Y. Sodium butyrate alleviates intestinal injury and microbial flora disturbance induced by lipopolysaccharides in rats. Food Funct. (2022) 13:1360–9. doi: 10.1039/D1FO03183J, PMID: 35044411

[103] Zhang Y Gu Y Ren H Wang S Zhong H Zhao X. Gut microbiome-related effects of berberine and probiotics on type 2 diabetes (the PREMOTE study). Nat Commun. (2020) 11:5015. doi: 10.1038/s41467-020-18414-8, PMID: 33024120 PMC7538905

[104] Cao H Li C Lei L Wang X Liu S Liu Q. Stachyose improves the effects of Berberine on glucose metabolism by regulating intestinal microbiota and short-chain fatty acids in spontaneous type 2 diabetic KKAy mice. Front Pharmacol. (2020) 11:578943. doi: 10.3389/fphar.2020.578943, PMID: 33192521 PMC7642818

[105] Neyrinck AM Sanchez CR Rodriguez J Cani PD Bindels LB Delzenne NM. Prebiotic effect of Berberine and curcumin is associated with the improvement of obesity in mice. Nutrients. (2021) 13:1436. doi: 10.3390/nu13051436, PMID: 33923174 PMC8145536

[106] Dong C Yu J Yang Y Zhang F Su W Fan Q. Berberine, a potential prebiotic to indirectly promote Akkermansia growth through stimulating gut mucin secretion. Biomed Pharmacother. (2021) 139:111595:111595. doi: 10.1016/j.biopha.2021.111595, PMID: 33862492

[107] Li X Yu M Zhu Z Lu C Jin M Rao Y. Oral delivery of infliximab using nano-in-microparticles for the treatment of inflammatory bowel disease. Carbohydr Polym. (2021) 273:118556:118556. doi: 10.1016/j.carbpol.2021.118556, PMID: 34560967

[108] Lima MSR de Lima VCO Piuvezam G de Azevedo KPM Maciel BLL Morais AHA. Mechanisms of action of anti-inflammatory proteins and peptides with anti-TNF-alpha activity and their effects on the intestinal barrier: a systematic review. PLoS One. (2022) 17:e0270749. doi: 10.1371/journal.pone.0270749, PMID: 35939430 PMC9359527

[109] Xiao YT Yan WH Cao Y Yan JK Cai W. Neutralization of IL-6 and TNF-alpha ameliorates intestinal permeability in DSS-induced colitis. Cytokine. (2016) 83:189–92. doi: 10.1016/j.cyto.2016.04.012, PMID: 27155817

[110] Patankar JV Muller TM Kantham S Acera MG Mascia F Scheibe K. E-type prostanoid receptor 4 drives resolution of intestinal inflammation by blocking epithelial necroptosis. Nat Cell Biol. (2021) 23:796–807. doi: 10.1038/s41556-021-00708-8, PMID: 34239062

[111] SantaCruz-Calvo S Bharath L Pugh G SantaCruz-Calvo L Lenin RR Lutshumba J. Adaptive immune cells shape obesity-associated type 2 diabetes mellitus and less prominent comorbidities. Nat Rev Endocrinol. (2022) 18:23–42. doi: 10.1038/s41574-021-00575-1, PMID: 34703027 PMC11005058

[112] Belizario JE Faintuch J Garay-Malpartida M. Gut microbiome dysbiosis and immunometabolism: new frontiers for treatment of metabolic diseases. Mediat Inflamm. (2018) 2018:2037838. doi: 10.1155/2018/2037838 PMC6304917 30622429

[113] Yang G Wei J Liu P Zhang Q Tian Y Hou G. Role of the gut microbiota in type 2 diabetes and related diseases. Metabolism. (2021) 117:154712:154712. doi: 10.1016/j.metabol.2021.154712 33497712

[114] Takiishi T Fenero CIM Camara NOS. Intestinal barrier and gut microbiota: shaping our immune responses throughout life. Tissue Barriers. (2017) 5:e1373208. doi: 10.1080/21688370.2017.1373208, PMID: 28956703 PMC5788425

[115] Pasini E Corsetti G Assanelli D Testa C Romano C Dioguardi FS. Effects of chronic exercise on gut microbiota and intestinal barrier in human with type 2 diabetes. Minerva Med. (2019) 110:3–11. doi: 10.23736/S0026-4806.18.05589-1, PMID: 30667205

[116] Sharma S Tripathi P. Gut microbiome and type 2 diabetes: where we are and where to go? J Nutr Biochem. (2019) 63:101–8. doi: 10.1016/j.jnutbio.2018.10.003, PMID: 30366260

[117] Salazar J Angarita L Morillo V Navarro C Martinez MS Chacin M. Microbiota and diabetes mellitus: role of lipid mediators. Nutrients. (2020) 12:3039. doi: 10.3390/nu12103039, PMID: 33023000 PMC7600362

[118] Han Y Wu L Ling Q Wu P Zhang C Jia L. Intestinal dysbiosis correlates with sirolimus-induced metabolic disorders in mice. Transplantation. (2021) 105:1017–29. doi: 10.1097/TP.0000000000003494, PMID: 33116044

[119] Zhou D Pan Q Shen F Cao HX Ding WJ Chen YW. Total fecal microbiota transplantation alleviates high-fat diet-induced steatohepatitis in mice via beneficial regulation of gut microbiota. Sci Rep. (2017) 7:1529:1529. doi: 10.1038/s41598-017-01751-y, PMID: 28484247 PMC5431549

[120] Delaune V Orci LA Lacotte S Peloso A Schrenzel J Lazarevic V. Fecal microbiota transplantation: a promising strategy in preventing the progression of non-alcoholic steatohepatitis and improving the anti-cancer immune response. Expert Opin Biol Ther. (2018) 18:1061–71. doi: 10.1080/14712598.2018.1518424, PMID: 30173562

[121] Machado D Barbosa JC Almeida D Andrade JC Freitas AC Gomes AM. Insights into the antimicrobial resistance profile of a next generation probiotic *Akkermansia muciniphila* DSM 22959. Int J Environ Res Public Health. (2022) 19:9152. doi: 10.3390/ijerph19159152, PMID: 35954507 PMC9367757

[122] Li S Qi C Zhu H Yu R Xie C Peng Y. *Lactobacillus reuteri* improves gut barrier function and affects diurnal variation of the gut microbiota in mice fed a high-fat diet. Food Funct. (2019) 10:4705–15. doi: 10.1039/C9FO00417C, PMID: 31304501

[123] Chen J Chen X Ho CL. Recent development of probiotic Bifidobacteria for treating human diseases. Front Bioeng Biotechnol. (2021) 9:770248. doi: 10.3389/fbioe.2021.770248, PMID: 35004640 PMC8727868

[124] Nie K Ma K Luo W Shen Z Yang Z Xiao M. Roseburia intestinalis: a beneficial gut organism from the discoveries in genus and species. Front Cell Infect Microbiol. (2021) 11:757718. doi: 10.3389/fcimb.2021.757718, PMID: 34881193 PMC8647967

[125] Saez-Lara MJ Robles-Sanchez C Ruiz-Ojeda FJ Plaza-Diaz J Gil A. Effects of probiotics and synbiotics on obesity, insulin resistance syndrome, type 2 diabetes and non-alcoholic fatty liver disease: a review of human clinical trials. Int J Mol Sci. (2016) 17:928. doi: 10.3390/ijms17060928, PMID: 27304953 PMC4926461

[126] Quigley EMM. Prebiotics and probiotics in digestive health. Clin Gastroenterol Hepatol. (2019) 17:333–44. doi: 10.1016/j.cgh.2018.09.028, PMID: 30267869

[127] Wang J Ji H Wang S Liu H Zhang W Zhang D. Probiotic lactobacillus plantarum promotes intestinal barrier function by strengthening the epithelium and modulating gut microbiota. Front Microbiol. (2018) 9:1953. doi: 10.3389/fmicb.2018.01953, PMID: 30197632 PMC6117384

[128] Wu H Xie S Miao J Li Y Wang Z Wang M. *Lactobacillus reuteri* maintains intestinal epithelial regeneration and repairs damaged intestinal mucosa. Gut Microbes. (2020) 11:997–1014. doi: 10.1080/19490976.2020.1734423, PMID: 32138622 PMC7524370

[129] Krumbeck JA Rasmussen HE Hutkins RW Clarke J Shawron K Keshavarzian A. Probiotic *Bifidobacterium* strains and galactooligosaccharides improve intestinal barrier function in obese adults but show no synergism when used together as synbiotics. Microbiome. (2018) 6:121:121. doi: 10.1186/s40168-018-0494-4, PMID: 29954454 PMC6022452

[130] Sergeev IN Aljutaily T Walton G Huarte E. Effects of synbiotic supplement on human gut microbiota, body composition and weight loss in obesity. Nutrients. (2020) 12:222. doi: 10.3390/nu12010222, PMID: 31952249 PMC7019807

[131] Depommier C Everard A Druart C Plovier H Van Hul M Vieira-Silva S. Supplementation with *Akkermansia muciniphila* in overweight and obese human volunteers: a proof-of-concept exploratory study. Nat Med. (2019) 25:1096–103. doi: 10.1038/s41591-019-0495-2, PMID: 31263284 PMC6699990

[132] Rodrigues VF Elias-Oliveira J Pereira IS Pereira JA Barbosa SC Machado MSG. *Akkermansia muciniphila* and gut immune system: a good friendship that attenuates inflammatory bowel disease, obesity, and diabetes. Front Immunol. (2022) 13:934695. doi: 10.3389/fimmu.2022.934695, PMID: 35874661 PMC9300896

[133] Rose EC Odle J Blikslager AT Ziegler AL. Probiotics, prebiotics and epithelial tight junctions: a promising approach to modulate intestinal barrier function. Int J Mol Sci. (2021) 22:6729. doi: 10.3390/ijms22136729, PMID: 34201613 PMC8268081

[134] Alvarez CS Badia J Bosch M Gimenez R Baldoma L. Outer membrane vesicles and soluble factors released by probiotic *Escherichia coli* Nissle 1917 and commensal ECOR63 enhance barrier function by regulating expression of tight junction proteins in intestinal epithelial cells. Front Microbiol. (2016) 7:1981. doi: 10.3389/fmicb.2016.01981, PMID: 28018313 PMC5156689

[135] Secher T Kassem S Benamar M Bernard I Boury M Barreau F. Oral administration of the probiotic strain *Escherichia coli* Nissle 1917 reduces susceptibility to neuroinflammation and repairs experimental autoimmune encephalomyelitis-induced intestinal barrier dysfunction. Front Immunol. (2017) 8:1096. doi: 10.3389/fimmu.2017.01096, PMID: 28959254 PMC5603654

[136] Fabrega MJ Rodriguez-Nogales A Garrido-Mesa J Algieri F Badia J Gimenez R. Intestinal anti-inflammatory effects of outer membrane vesicles from *Escherichia coli* Nissle 1917 in DSS-experimental colitis in mice. Front Microbiol. (2017) 8:1274. doi: 10.3389/fmicb.2017.01274, PMID: 28744268 PMC5504144

[137] Zhou J Li M Chen Q Li X Chen L Dong Z. Programmable probiotics modulate inflammation and gut microbiota for inflammatory bowel disease treatment after effective oral delivery. Nat Commun. (2022) 13:3432:3432. doi: 10.1038/s41467-022-31171-0, PMID: 35701435 PMC9198027

[138] Liu Q Yu Z Tian F Zhao J Zhang H Zhai Q. Surface components and metabolites of probiotics for regulation of intestinal epithelial barrier. Microb Cell Factories. (2020) 19:23:23. doi: 10.1186/s12934-020-1289-4, PMID: 32024520 PMC7003451

[139] Praveschotinunt P Duraj-Thatte AM Gelfat I Bahl F Chou DB Joshi NS. Engineered *E. coli* Nissle 1917 for the delivery of matrix-tethered therapeutic domains to the gut. Nat Commun. (2019) 10:5580:5580. doi: 10.1038/s41467-019-13336-6, PMID: 31811125 PMC6898321

[140] Yu M Kim J Ahn JH Moon Y. Nononcogenic restoration of the intestinal barrier by *E. coli*-delivered human EGF. JCI. Insight. (2019) 4:e125166. doi: 10.1172/jci.insight.125166, PMID: 31434808 PMC6777819

[141] Tian P Li B He C Song W Hou A Tian S. Antidiabetic (type 2) effects of *Lactobacillus* G15 and Q14 in rats through regulation of intestinal permeability and microbiota. Food Funct. (2016) 7:3789–97. doi: 10.1039/C6FO00831C, PMID: 27713957

[142] Caballero-Franco C Keller K De Simone C Chadee K. The VSL#3 probiotic formula induces mucin gene expression and secretion in colonic epithelial cells. Am J Physiol Gastrointest Liver Physiol. (2007) 292:G315–22. doi: 10.1152/ajpgi.00265.2006 16973917

[143] Engevik MA Luk B Chang-Graham AL Hall A Herrmann B Ruan W. Bifidobacterium dentium fortifies the intestinal mucus layer via autophagy and calcium signaling pathways. mBio. (2019) 10:e01087-19. doi: 10.1128/mBio.01087-19, PMID: 31213556 PMC6581858

[144] Mennigen R Nolte K Rijcken E Utech M Loeffler B Senninger N. Probiotic mixture VSL#3 protects the epithelial barrier by maintaining tight junction protein expression and preventing apoptosis in a murine model of colitis. Am J Physiol Gastrointest Liver Physiol. (2009) 296:G1140–9. doi: 10.1152/ajpgi.90534.2008 19221015

[145] Yang X Yang J Ye Z Zhang G Nie W Cheng H. Physiologically inspired mucin coated *Escherichia coli* Nissle 1917 enhances biotherapy by regulating the pathological microenvironment to improve intestinal colonization. ACS Nano. (2022) 16:4041–58. doi: 10.1021/acsnano.1c09681, PMID: 35230097

[146] Li Y Liu T Yan C Xie R Guo Z Wang S. Diammonium glycyrrhizinate protects against nonalcoholic fatty liver disease in mice through modulation of gut microbiota and restoration of intestinal barrier. Mol Pharm. (2018) 15:3860–70. doi: 10.1021/acs.molpharmaceut.8b00347, PMID: 30036479

[147] Xu W Lin L Liu A Zhang T Zhang S Li Y. L-Theanine affects intestinal mucosal immunity by regulating short-chain fatty acid metabolism under dietary fiber feeding. Food Funct. (2020 Sep 23) 11:8369–79. doi: 10.1039/D0FO01069C, PMID: 32935679

[148] Younossi ZM Ratziu V Loomba R Rinella M Anstee QM Goodman Z. Obeticholic acid for the treatment of non-alcoholic steatohepatitis: interim analysis from a multicentre, randomised, placebo-controlled phase 3 trial. Lancet. (2019) 394:2184–96. doi: 10.1016/S0140-6736(19)33041-7, PMID: 31813633

[149] Zhang DY Zhu L Liu HN Tseng YJ Weng SQ Liu TT. The protective effect and mechanism of the FXR agonist obeticholic acid via targeting gut microbiota in non-alcoholic fatty liver disease. Drug Des Devel Ther. (2019) 13:2249–70. doi: 10.2147/DDDT.S207277, PMID: 31308634 PMC6617567

[150] Zhang N Tao J Gao L Bi Y Li P Wang H. Liraglutide attenuates nonalcoholic fatty liver disease by modulating gut microbiota in rats administered a high-fat diet. Biomed Res Int. (2020) 2020:1–10. doi: 10.1155/2020/2947549, PMID: 32149099 PMC7049398

[151] Yang S Hu T Liu H Lv YL Zhang W Li H. Akebia saponin D ameliorates metabolic syndrome (MetS) via remodeling gut microbiota and attenuating intestinal barrier injury. Biomed Pharmacother. (2021) 138:111441:111441. doi: 10.1016/j.biopha.2021.111441, PMID: 33652261

[152] Chang CJ Lin CS Lu CC Martel J Ko YF Ojcius DM. Ganoderma lucidum reduces obesity in mice by modulating the composition of the gut microbiota. Nat Commun. (2015) 6:7489. doi: 10.1038/ncomms8489, PMID: 26102296 PMC4557287

[153] Sang T Guo C Guo D Wu J Wang Y Wang Y. Suppression of obesity and inflammation by polysaccharide from sporoderm-broken spore of Ganoderma lucidum via gut microbiota regulation. Carbohydr Polym. (2021) 256:117594:117594. doi: 10.1016/j.carbpol.2020.117594, PMID: 33483079

[154] Federici S Kredo-Russo S Valdes-Mas R Kviatcovsky D Weinstock E Matiuhin Y. Targeted suppression of human IBD-associated gut microbiota commensals by phage consortia for treatment of intestinal inflammation. Cells. (2022) 185:2879–2898.e24. doi: 10.1016/j.cell.2022.07.003, PMID: 35931020

[155] Yang Y Du H Zou G Song Z Zhou Y Li H. Encapsulation and delivery of phage as a novel method for gut flora manipulation in situ: a review. J Control Release. (2022) 353:634–49. doi: 10.1016/j.jconrel.2022.11.048, PMID: 36464065

[156] Shuwen H Kefeng D. Intestinal phages interact with bacteria and are involved in human diseases. Gut Microbes. (2022) 14:2113717. doi: 10.1080/19490976.2022.2113717, PMID: 36037202 PMC9427043

[157] Zhao H Li Y Lv P Huang J Tai R Jin X. Salmonella phages affect the intestinal barrier in chicks by altering the composition of early intestinal flora: association with time of phage use. Front Microbiol. (2022) 13:947640. doi: 10.3389/fmicb.2022.947640, PMID: 35910610 PMC9329052

[158] Chen PB Black AS Sobel AL Zhao Y Mukherjee P Molparia B. Directed remodeling of the mouse gut microbiome inhibits the development of atherosclerosis. Nat Biotechnol. (2020) 38:1288–97. doi: 10.1038/s41587-020-0549-5, PMID: 32541956 PMC7641989

[159] Dong X Pan P Zheng DW Bao P Zeng X Zhang XZ. Bioinorganic hybrid bacteriophage for modulation of intestinal microbiota to remodel tumor-immune microenvironment against colorectal cancer. Sci Adv. (2020) 6:eaba1590. doi: 10.1126/sciadv.aba1590, PMID: 32440552 PMC7228756

[160] Baaziz H Baker ZR Franklin HC Hsu BB. Rehabilitation of a misbehaving microbiome: phages for the remodeling of bacterial composition and function. iScience. (2022) 25:104146:104146. doi: 10.1016/j.isci.2022.104146, PMID: 35402871 PMC8991392

[161] Weingarden AR Vaughn BP. Intestinal microbiota, fecal microbiota transplantation, and inflammatory bowel disease. Gut Microbes. (2017) 8:238–52. doi: 10.1080/19490976.2017.1290757, PMID: 28609251 PMC5479396

[162] Antushevich H. Fecal microbiota transplantation in disease therapy. Clin Chim Acta. (2020) 503:90–8. doi: 10.1016/j.cca.2019.12.010, PMID: 31968211

[163] Vrieze A Van Nood E Holleman F Salojarvi J Kootte RS Bartelsman JF. Transfer of intestinal microbiota from lean donors increases insulin sensitivity in individuals with metabolic syndrome. Gastroenterology. (2012) 143:e7:913–916.e7. doi: 10.1053/j.gastro.2012.06.031, PMID: 22728514

[164] Kootte RS Levin E Salojarvi J Smits LP Hartstra AV Udayappan SD. Improvement of insulin sensitivity after lean donor feces in metabolic syndrome is driven by baseline intestinal microbiota composition. Cell Metab. (2017) 26:e6:611–619.e6. doi: 10.1016/j.cmet.2017.09.008, PMID: 28978426

[165] Xie WR Yang XY Deng ZH Zheng YM Zhang R Wu LH. Effects of washed microbiota transplantation on serum uric acid levels, symptoms, and intestinal barrier function in patients with acute and recurrent gout: a pilot study. Dig Dis. (2022) 40:684–90. doi: 10.1159/000521273, PMID: 34872097

[166] Bidu C Escoula Q Bellenger S Spor A Galan M Geissler A. The transplantation of omega3 PUFA-altered gut microbiota of fat-1 mice to wild-type littermates prevents obesity and associated metabolic disorders. Diabetes. (2018) 67:1512–23. doi: 10.2337/db17-1488, PMID: 29793999

[167] Assimakopoulos SF Papadopoulou I Bantouna D de Lastic AL Rodi M Mouzaki A. Fecal microbiota transplantation and hydrocortisone ameliorate intestinal barrier dysfunction and improve survival in a rat model of cecal ligation and puncture-induced sepsis. Shock. (2021) 55:666–75. doi: 10.1097/SHK.0000000000001566, PMID: 32496421

[168] Rao J Xie R Lin L Jiang J Du L Zeng X. Fecal microbiota transplantation ameliorates gut microbiota imbalance and intestinal barrier damage in rats with stress-induced depressive-like behavior. Eur J Neurosci. (2021) 53:3598–611. doi: 10.1111/ejn.15192, PMID: 33742731

[169] Gai X Wang H Li Y Zhao H He C Wang Z. Fecal microbiota transplantation protects the intestinal mucosal barrier by reconstructing the gut microbiota in a murine model of sepsis. Front Cell Infect Microbiol. (2021) 11:736204. doi: 10.3389/fcimb.2021.736204, PMID: 34631604 PMC8493958

[170] Zhao Z Ning J Bao XQ Shang M Ma J Li G. Fecal microbiota transplantation protects rotenone-induced Parkinson's disease mice via suppressing inflammation mediated by the lipopolysaccharide-TLR4 signaling pathway through the microbiota-gut-brain axis. Microbiome. (2021) 9:226. doi: 10.1186/s40168-021-01107-9, PMID: 34784980 PMC8597301

[171] Cheng S Ma X Geng S Jiang X Li Y Hu L. Fecal microbiota transplantation beneficially regulates intestinal mucosal autophagy and alleviates gut barrier injury. mSystems. (2018) 3:e00137-18. doi: 10.1128/mSystems.00137-18, PMID: 30320222 PMC6178585

[172] Zellmer C Sater MRA Huntley MH Osman M Olesen SW Ramakrishna B. Shiga toxin-producing *Escherichia coli* transmission via fecal microbiota transplant. Clin Infect Dis. (2021) 72:e876–80. doi: 10.1093/cid/ciaa1486, PMID: 33159210

[173] Ott SJ Waetzig GH Rehman A Moltzau-Anderson J Bharti R Grasis JA. Efficacy of sterile fecal filtrate transfer for treating patients with *Clostridium difficile* infection. Gastroenterology. (2017) 152:799–811.e7. doi: 10.1053/j.gastro.2016.11.010, PMID: 27866880

[174] Zhang T Lu G Zhao Z Liu Y Shen Q Li P. Washed microbiota transplantation vs. manual fecal microbiota transplantation: clinical findings, animal studies and in vitro screening. Protein Cell. (2020) 11:251–66. doi: 10.1007/s13238-019-00684-8, PMID: 31919742 PMC7093410

[175] Weston KS Wisloff U Coombes JS. High-intensity interval training in patients with lifestyle-induced cardiometabolic disease: a systematic review and meta-analysis. Br J Sports Med. (2014) 48:1227–34. doi: 10.1136/bjsports-2013-092576, PMID: 24144531

[176] Guzman A Navarro E Obando L Pacheco J Quiros K Vasquez L. Effectiveness of interventions for the reversal of a metabolic syndrome diagnosis: an update of a meta-analysis of mixed treatment comparison studies. Biomedica. (2019) 39:647–62. doi: 10.7705/biomedica.4684, PMID: 31860177 PMC7363343

[177] Perdomo CM Fruhbeck G Escalada J. Impact of nutritional changes on nonalcoholic fatty liver disease. Nutrients. (2019) 11:677. doi: 10.3390/nu11030677, PMID: 30901929 PMC6470750

[178] Li M Xu Y Wan Q Shen F Xu M Zhao Z. Individual and combined associations of modifiable lifestyle and metabolic health status with new-onset diabetes and major cardiovascular events: the China cardiometabolic disease and cancer cohort (4C) study. Diabetes Care. (2020) 43:1929–36. doi: 10.2337/dc20-0256, PMID: 32540923

[179] Gabriel BM Zierath JR. Circadian rhythms and exercise - re-setting the clock in metabolic disease. Nat Rev Endocrinol. (2019) 15:197–206. doi: 10.1038/s41574-018-0150-x, PMID: 30655625

[180] Pedersen BK Saltin B. Exercise as medicine - evidence for prescribing exercise as therapy in 26 different chronic diseases. Scand J Med Sci Sports. (2015) 25:1–72. doi: 10.1111/sms.12581, PMID: 26606383

[181] Stefani L Galanti G. Physical exercise prescription in metabolic chronic disease. Adv Exp Med Biol. (2017) 1005:123–41. doi: 10.1007/978-981-10-5717-5_6, PMID: 28916931

[182] Tjonna AE Lee SJ Rognmo O Stolen TO Bye A Haram PM. Aerobic interval training versus continuous moderate exercise as a treatment for the metabolic syndrome: a pilot study. Circulation. (2008) 118:346–54. doi: 10.1161/CIRCULATIONAHA.108.772822, PMID: 18606913 PMC2777731

[183] Bacchi E Negri C Zanolin ME Milanese C Faccioli N Trombetta M. Metabolic effects of aerobic training and resistance training in type 2 diabetic subjects: a randomized controlled trial (the RAED2 study). Diabetes Care. (2012) 35:676–82. doi: 10.2337/dc11-1655, PMID: 22344613 PMC3308269

[184] Sabag A Way KL Sultana RN Keating SE Gerofi JA Chuter VH. The effect of a novel low-volume aerobic exercise intervention on liver fat in type 2 diabetes: a randomized controlled trial. Diabetes Care. (2020) 43:2371–8. doi: 10.2337/dc19-2523, PMID: 32732374

[185] O'Gorman P Naimimohasses S Monaghan A Kennedy M Melo AM Ni Fhloinn D. Improvement in histological endpoints of MAFLD following a 12-week aerobic exercise intervention. Aliment Pharmacol Ther. (2020 Oct) 52:1387–98. doi: 10.1111/apt.15989, PMID: 32717123

[186] Carbajo-Pescador S Porras D Garcia-Mediavilla MV Martinez-Florez S Juarez-Fernandez M Cuevas MJ. Beneficial effects of exercise on gut microbiota functionality and barrier integrity, and gut-liver crosstalk in an in vivo model of early obesity and non-alcoholic fatty liver disease. Dis Model Mech. (2019) 12:dmm039206. doi: 10.1242/dmm.039206, PMID: 30971408 PMC6550047

[187] Wang J Zhang Q Xia J Sun H. Moderate treadmill exercise modulates gut microbiota and improves intestinal barrier in high-fat-diet-induced obese mice via the AMPK/CDX2 signaling pathway. Diabetes Metab Syndr Obes. (2022) 15:209–23. doi: 10.2147/DMSO.S346007, PMID: 35087282 PMC8789310

[188] Feng V Bawa KK Marzolini S Kiss A Oh P Herrmann N. Impact of 12-week exercise program on biomarkers of gut barrier integrity in patients with coronary artery disease. PLoS One. (2021) 16:e0260165. doi: 10.1371/journal.pone.0260165, PMID: 34797867 PMC8604291

[189] Shin HE Kwak SE Zhang DD Lee J Yoon KJ Cho HS. Effects of treadmill exercise on the regulation of tight junction proteins in aged mice. Exp Gerontol. (2020) 141:111077:111077. doi: 10.1016/j.exger.2020.111077, PMID: 32898618

[190] Li K Liu A Zong W Dai L Liu Y Luo R. Moderate exercise ameliorates osteoarthritis by reducing lipopolysaccharides from gut microbiota in mice. Saudi J Biol Sci. (2021) 28:40–9. doi: 10.1016/j.sjbs.2020.08.027, PMID: 33424281 PMC7783636

[191] Monda V Villano I Messina A Valenzano A Esposito T Moscatelli F. Exercise modifies the gut microbiota with positive health effects. Oxidative Med Cell Longev. (2017) 2017:1–8. doi: 10.1155/2017/3831972, PMID: 28357027 PMC5357536

[192] Jayedi A Emadi A Shab-Bidar S. Dose-dependent effect of supervised aerobic exercise on HbA(1c) in patients with type 2 diabetes: a meta-analysis of randomized controlled trials. Sports Med. (2022) 52:1919–38. doi: 10.1007/s40279-022-01673-4, PMID: 35362859

[193] Haganes KL Silva CP Eyjolfsdottir SK Steen S Grindberg M Lydersen S. Time-restricted eating and exercise training improve HbA1c and body composition in women with overweight/obesity: a randomized controlled trial. Cell Metab. (2022) 34:e4:1457–1471.e4. doi: 10.1016/j.cmet.2022.09.003, PMID: 36198292

[194] Dupuit M Chavanelle V Chassaing B Perriere F Etienne M Plissonneau C. The TOTUM-63 supplement and high-intensity interval training combination limits weight gain, improves glycemic control, and influences the composition of gut mucosa-associated bacteria in rats on a high fat diet. Nutrients. (2021) 13:1569. doi: 10.3390/nu13051569, PMID: 34066988 PMC8151333

[195] Nolan CJ Prentki M. Insulin resistance and insulin hypersecretion in the metabolic syndrome and type 2 diabetes: time for a conceptual framework shift. Diab Vasc Dis Res. (2019) 16:118–27. doi: 10.1177/1479164119827611, PMID: 30770030

[196] Malesza IJ Malesza M Walkowiak J Mussin N Walkowiak D Aringazina R. High-fat, western-style diet, systemic inflammation, and gut microbiota: a narrative review. Cells. (2021) 10:3164. doi: 10.3390/cells10113164, PMID: 34831387 PMC8619527

[197] Riccardi G Vaccaro O Costabile G Rivellese AA. How well can we control dyslipidemias through lifestyle modifications? Curr Cardiol Rep. (2016 Jul) 18:66. doi: 10.1007/s11886-016-0744-7 27216846

[198] Martinez-Lopez S Sarria B Sierra-Cinos JL Goya L Mateos R Bravo L. Realistic intake of a flavanol-rich soluble cocoa product increases HDL-cholesterol without inducing anthropometric changes in healthy and moderately hypercholesterolemic subjects. Food Funct. (2014) 5:364–74. doi: 10.1039/c3fo60352k, PMID: 24394704

[199] Bernardi S Del Bo C Marino M Gargari G Cherubini A Andres-Lacueva C. Polyphenols and intestinal permeability: rationale and future perspectives. J Agric Food Chem. (2020) 68:1816–29. doi: 10.1021/acs.jafc.9b02283, PMID: 31265272

[200] Li W Yang H Zhao Q Wang X Zhang J Zhao X. Polyphenol-rich loquat fruit extract prevents fructose-induced nonalcoholic fatty liver disease by modulating glycometabolism, lipometabolism, oxidative stress, inflammation, intestinal barrier, and gut microbiota in mice. J Agric Food Chem. (2019) 67:7726–37. doi: 10.1021/acs.jafc.9b02523, PMID: 31203627

[201] Wang K Jin X Chen Y Song Z Jiang X Hu F. Polyphenol-rich propolis extracts strengthen intestinal barrier function by activating AMPK and ERK signaling. Nutrients. (2016) 8:272. doi: 10.3390/nu8050272, PMID: 27164138 PMC4882685

[202] Del Bo C Bernardi S Cherubini A Porrini M Gargari G Hidalgo-Liberona N. A polyphenol-rich dietary pattern improves intestinal permeability, evaluated as serum zonulin levels, in older subjects: the MaPLE randomised controlled trial. Clin Nutr. (2021) 40:3006–18. doi: 10.1016/j.clnu.2020.12.014, PMID: 33388204

[203] Hidalgo-Liberona N Gonzalez-Dominguez R Vegas E Riso P Del Bo C Bernardi S. Increased intestinal permeability in older subjects impacts the beneficial effects of dietary polyphenols by modulating their bioavailability. J Agric Food Chem. (2020) 68:12476–84. doi: 10.1021/acs.jafc.0c04976, PMID: 33084335

[204] Evans CEL. Dietary fibre and cardiovascular health: a review of current evidence and policy. Proc Nutr Soc. (2020) 79:61–7. doi: 10.1017/S0029665119000673, PMID: 31266545

[205] Xie Y Gou L Peng M Zheng J Chen L. Effects of soluble fiber supplementation on glycemic control in adults with type 2 diabetes mellitus: a systematic review and meta-analysis of randomized controlled trials. Clin Nutr. (2021) 40:1800–10. doi: 10.1016/j.clnu.2020.10.032, PMID: 33162192

[206] Partula V Deschasaux M Druesne-Pecollo N Latino-Martel P Desmetz E Chazelas E. Associations between consumption of dietary fibers and the risk of cardiovascular diseases, cancers, type 2 diabetes, and mortality in the prospective NutriNet-Sante cohort. Am J Clin Nutr. (2020) 112:195–207. doi: 10.1093/ajcn/nqaa063, PMID: 32369545

[207] Desai MS Seekatz AM Koropatkin NM Kamada N Hickey CA Wolter M. A dietary fiber-deprived gut microbiota degrades the colonic mucus barrier and enhances pathogen susceptibility. Cells. (2016) 167:1339–1353.e21. doi: 10.1016/j.cell.2016.10.043, PMID: 27863247 PMC5131798

[208] Monk JM Wu W Lepp D Wellings HR Hutchinson AL Liddle DM. Navy bean supplemented high-fat diet improves intestinal health, epithelial barrier integrity and critical aspects of the obese inflammatory phenotype. J Nutr Biochem. (2019) 70:91–104. doi: 10.1016/j.jnutbio.2019.04.009, PMID: 31195365

[209] Paone P Suriano F Jian C Korpela K Delzenne NM Van Hul M. Prebiotic oligofructose protects against high-fat diet-induced obesity by changing the gut microbiota, intestinal mucus production, glycosylation and secretion. Gut Microbes. (2022) 14:2152307. doi: 10.1080/19490976.2022.2152307, PMID: 36448728 PMC9715274

[210] Wang H He C Liu Y Zhao H Long L Gai X. Soluble dietary fiber protects intestinal mucosal barrier by improving intestinal flora in a murine model of sepsis. Biomed Pharmacother. (2020) 129:110343. doi: 10.1016/j.biopha.2020.110343, PMID: 32593968

[211] Hu ED Chen DZ Wu JL Lu FB Chen L Zheng MH. High fiber dietary and sodium butyrate attenuate experimental autoimmune hepatitis through regulation of immune regulatory cells and intestinal barrier. Cell Immunol. (2018) 328:24–32. doi: 10.1016/j.cellimm.2018.03.003, PMID: 29627063

[212] Zeng X Xing X Gupta M Keber FC Lopez JG Lee YJ. Gut bacterial nutrient preferences quantified in vivo. Cells. (2022) 185:3441–3456.e19. doi: 10.1016/j.cell.2022.07.020, PMID: 36055202 PMC9450212

[213] Nie Q Hu J Gao H Li M Sun Y Chen H. Bioactive dietary fibers selectively promote gut microbiota to exert antidiabetic effects. J Agric Food Chem. (2021) 69:7000–15. doi: 10.1021/acs.jafc.1c01465, PMID: 34139119

[214] Makki K Brolin H Petersen N Henricsson M Christensen DP Khan MT. 6alpha-hydroxylated bile acids mediate TGR5 signalling to improve glucose metabolism upon dietary fiber supplementation in mice. Gut. (2023) 72:314–24. doi: 10.1136/gutjnl-2021-326541, PMID: 35697422 PMC9872241

[215] Zhai Z Dong W Sun Y Gu Y Ma J Wang B. Vitamin-microbiota crosstalk in intestinal inflammation and carcinogenesis. Nutrients. (2022) 14:3383. doi: 10.3390/nu14163383, PMID: 36014889 PMC9414212

[216] Lips P Eekhoff M van Schoor N Oosterwerff M de Jongh R Krul-Poel Y. Vitamin D and type 2 diabetes. J Steroid Biochem Mol Biol. (2017) 173:280–5. doi: 10.1016/j.jsbmb.2016.11.021, PMID: 27932304

[217] Blaner WS. Vitamin a signaling and homeostasis in obesity, diabetes, and metabolic disorders. Pharmacol Ther. (2019) 197:153–78. doi: 10.1016/j.pharmthera.2019.01.006, PMID: 30703416 PMC6520171

[218] Linsalata M Riezzo G Orlando A D'Attoma B Prospero L Tutino V. The relationship between low serum vitamin D levels and altered intestinal barrier function in patients with IBS diarrhoea undergoing a Long-term low-FODMAP diet: novel observations from a clinical trial. Nutrients. (2021) 13:1011. doi: 10.3390/nu13031011, PMID: 33801020 PMC8004066

[219] He C Deng J Hu X Zhou S Wu J Xiao D. Vitamin a inhibits the action of LPS on the intestinal epithelial barrier function and tight junction proteins. Food Funct. (2019) 10:1235–42. doi: 10.1039/C8FO01123K, PMID: 30747184

[220] Dong S Singh TP Wei X Yao H Wang H. Protective effect of 1,25-Dihydroxy vitamin D3 on pepsin-trypsin-resistant gliadin-induced tight junction injuries. Dig Dis Sci. (2018) 63:92–104. doi: 10.1007/s10620-017-4738-0, PMID: 28871457

[221] Du J Chen Y Shi Y Liu T Cao Y Tang Y. 1,25-Dihydroxyvitamin D protects intestinal epithelial barrier by regulating the myosin light chain kinase signaling pathway. Inflamm Bowel Dis. (2015) 21:2495–506. doi: 10.1097/MIB.0000000000000526, PMID: 26287999 PMC4646414

[222] Cheng J Balbuena E Miller B Eroglu A. The role of beta-carotene in colonic inflammation and intestinal barrier integrity. Front Nutr. (2021) 8:723480. doi: 10.3389/fnut.2021.723480, PMID: 34646849 PMC8502815

[223] Barbalho SM Goulart RA Gasparini RG. Associations between inflammatory bowel diseases and vitamin D. Crit Rev Food Sci Nutr. (2019) 59:1347–56. doi: 10.1080/10408398.2017.1406333 29236523

[224] Kuang H Ma Y Liu Y. Protective effect of beta-carotene on OVA-induced food allergy in mice by strengthening intestinal epithelial barrier function and regulating intestinal microflora. Food Funct. (2022) 13:12330–41. doi: 10.1039/D2FO02272A, PMID: 36354054

[225] Pang B Jin H Liao N Li J Jiang C Shi J. Vitamin A supplementation ameliorates ulcerative colitis in gut microbiota-dependent manner. Food Res Int. (2021) 148:110568. doi: 10.1016/j.foodres.2021.110568, PMID: 34507723

[226] Ge Y Zadeh M Mohamadzadeh M. Vitamin B12 regulates the transcriptional, metabolic, and epigenetic programing in human ileal epithelial cells. Nutrients. (2022) 14:2825. doi: 10.3390/nu14142825, PMID: 35889782 PMC9321803

[227] Harvie MN Pegington M Mattson MP Frystyk J Dillon B Evans G. The effects of intermittent or continuous energy restriction on weight loss and metabolic disease risk markers: a randomized trial in young overweight women. Int J Obes. (2011) 35:714–27. doi: 10.1038/ijo.2010.171, PMID: 20921964 PMC3017674

[228] Di W Lv Y Xia F Sheng Y Liu J Ding G. Improvement of intestinal stem cells and barrier function via energy restriction in middle-aged C57BL/6 mice. Nutr Res. (2020) 81:47–57. doi: 10.1016/j.nutres.2020.06.015, PMID: 32877836

[229] Akagi K Wilson KA Katewa SD Ortega M Simons J Hilsabeck TA. Dietary restriction improves intestinal cellular fitness to enhance gut barrier function and lifespan in *D. melanogaster*. PLoS Genet. (2018) 14:e1007777. doi: 10.1371/journal.pgen.1007777, PMID: 30383748 PMC6233930

[230] Seimon RV Wild-Taylor AL Keating SE McClintock S Harper C Gibson AA. Effect of weight loss via severe vs moderate energy restriction on lean mass and body composition among postmenopausal women with obesity: the TEMPO diet randomized clinical trial. JAMA Netw Open. (2019) 2:e1913733. doi: 10.1001/jamanetworkopen.2019.13733, PMID: 31664441 PMC6824325

[231] Mardon J Habauzit V Trzeciakiewicz A Davicco MJ Lebecque P Mercier S. Influence of high and low protein intakes on age-related bone loss in rats submitted to adequate or restricted energy conditions. Calcif Tissue Int. (2008) 82:373–82. doi: 10.1007/s00223-008-9125-6, PMID: 18437274

[232] Li M Wang S Li Y Zhao M Kuang J Liang D. Gut microbiota-bile acid crosstalk contributes to the rebound weight gain after calorie restriction in mice. Nat Commun. (2022) 13:2060. doi: 10.1038/s41467-022-29589-7, PMID: 35440584 PMC9018700

[233] Mattson MP Longo VD Harvie M. Impact of intermittent fasting on health and disease processes. Ageing Res Rev. (2017) 39:46–58. doi: 10.1016/j.arr.2016.10.005, PMID: 27810402 PMC5411330

[234] Tuganbaev T Mor U Bashiardes S Liwinski T Nobs SP Leshem A. Diet diurnally regulates small intestinal microbiome-epithelial-immune homeostasis and enteritis. Cells. (2020) 182:e21:1441–1459.e21. doi: 10.1016/j.cell.2020.08.027, PMID: 32888430

[235] Vidmar AP Naguib M Raymond JK Salvy SJ Hegedus E Wee CP. Time-limited eating and continuous glucose monitoring in adolescents with obesity: a pilot study. Nutrients. (2021) 13:3697. doi: 10.3390/nu13113697, PMID: 34835953 PMC8624400

[236] Sutton EF Beyl R Early KS Cefalu WT Ravussin E Peterson CM. Early time-restricted feeding improves insulin sensitivity, blood pressure, and oxidative stress even without weight loss in men with prediabetes. Cell Metab. (2018) 27:1212–1221.e3. doi: 10.1016/j.cmet.2018.04.010, PMID: 29754952 PMC5990470

[237] Wilkinson MJ Manoogian ENC Zadourian A Lo H Fakhouri S Shoghi A. Ten-hour time-restricted eating reduces weight, blood pressure, and atherogenic lipids in patients with metabolic syndrome. Cell Metab. (2020) 31:92–104.e5. doi: 10.1016/j.cmet.2019.11.004, PMID: 31813824 PMC6953486

[238] Carter S Clifton PM Keogh JB. Effect of intermittent compared with continuous energy restricted diet on glycemic control in patients with type 2 diabetes: a randomized noninferiority trial. JAMA Netw Open. (2018) 1:e180756. doi: 10.1001/jamanetworkopen.2018.0756, PMID: 30646030 PMC6324303

[239] Bishehsari F Engen PA Adnan D Sarrafi S Wilber S Shaikh M. Abnormal food timing and predisposition to weight gain: role of barrier dysfunction and microbiota. Transl Res. (2021) 231:113–23. doi: 10.1016/j.trsl.2020.11.007, PMID: 33221482 PMC8016699

[240] Hepler C Weidemann BJ Waldeck NJ Marcheva B Cedernaes J Thorne AK. Time-restricted feeding mitigates obesity through adipocyte thermogenesis. Science. (2022) 378:276–84. doi: 10.1126/science.abl8007, PMID: 36264811 PMC10150371

[241] Tinkum KL Stemler KM White LS Loza AJ Jeter-Jones S Michalski BM. Fasting protects mice from lethal DNA damage by promoting small intestinal epithelial stem cell survival. Proc Natl Acad Sci U S A. (2015) 112:E7148–54. doi: 10.1073/pnas.1509249112, PMID: 26644583 PMC4697381

[242] Frazier K Kambal A Zale EA Pierre JF Hubert N Miyoshi S. High-fat diet disrupts REG3gamma and gut microbial rhythms promoting metabolic dysfunction. Cell Host Microbe. (2022) 30:809–823.e6. doi: 10.1016/j.chom.2022.03.030, PMID: 35439436 PMC9281554

[243] Seillet C Luong K Tellier J Jacquelot N Shen RD Hickey P. Author correction: the neuropeptide VIP confers anticipatory mucosal immunity by regulating ILC3 activity. Nat Immunol. (2020) 21:354. doi: 10.1038/s41590-020-0606-8, PMID: 32001823

[244] Coleman KJ Wellman R Fitzpatrick SL Conroy MB Hlavin C Lewis KH. Comparative safety and effectiveness of roux-en-Y gastric bypass and sleeve gastrectomy for weight loss and type 2 diabetes across race and ethnicity in the PCORnet bariatric study cohort. JAMA Surg. (2022) 157:897–906. doi: 10.1001/jamasurg.2022.3714, PMID: 36044239 PMC9434478

[245] Murphy R Plank LD Clarke MG Evennett NJ Tan J Kim DDW. Effect of banded roux-en-Y gastric bypass versus sleeve gastrectomy on diabetes remission at 5 years among patients with obesity and type 2 diabetes: a blinded randomized clinical trial. Diabetes Care. (2022) 45:1503–11. doi: 10.2337/dc21-2498, PMID: 35554515 PMC9274222

[246] Hanipah ZN Schauer PR. Bariatric surgery as a long-term treatment for type 2 diabetes/metabolic syndrome. Annu Rev Med. (2020) 71:1–15. doi: 10.1146/annurev-med-053117-123246, PMID: 31986081

[247] Buchwald H. The evolution of metabolic/bariatric surgery. Obes Surg. (2014) 24:1126–35. doi: 10.1007/s11695-014-1354-3, PMID: 25008469

[248] Wilbrink J Bernards N Mujagic Z van Avesaat M Pijls K Klaassen T. Intestinal barrier function in morbid obesity: results of a prospective study on the effect of sleeve gastrectomy. Int J Obes. (2020) 44:368–76. doi: 10.1038/s41366-019-0492-z, PMID: 31819200

[249] Guo Y Liu CQ Liu GP Huang ZP Zou DJ. Roux-en-Y gastric bypass decreases endotoxemia and inflammatory stress in association with improvements in gut permeability in obese diabetic rats. J Diabetes. (2019) 11:786–93. doi: 10.1111/1753-0407.12906, PMID: 30714321

[250] Iwaniak P Tomaszewska E Muszynski S Marszalek-Grabska M Pierzynowski SG Dobrowolski P. Dietary alpha-ketoglutarate partially abolishes adverse changes in the small intestine after gastric bypass surgery in a rat model. Nutrients. (2022) 14:2062. doi: 10.3390/nu14102062, PMID: 35631203 PMC9146360

[251] Casselbrant A Elias E Fandriks L Wallenius V. Expression of tight-junction proteins in human proximal small intestinal mucosa before and after roux-en-Y gastric bypass surgery. Surg Obes Relat Dis. (2015) 11:45–53. doi: 10.1016/j.soard.2014.05.009, PMID: 25264329

[252] Chaudhari SN Harris DA Aliakbarian H Luo JN Henke MT Subramaniam R. Bariatric surgery reveals a gut-restricted TGR5 agonist with anti-diabetic effects. Nat Chem Biol. (2021) 17:20–9. doi: 10.1038/s41589-020-0604-z, PMID: 32747812 PMC7891870

[253] Cazzo E Gestic MA Utrini MP Chaim FD Geloneze B Pareja JC. Glp-2: a poorly understood mediator enrolled in various bariatric/metabolic surgery-related pathophysiologic mechanisms. Arq Bras Cir Dig. (2016) 29:272–5. doi: 10.1590/0102-6720201600040014, PMID: 28076485 PMC5225870

[254] Ning MM Yang WJ Guan WB Gu YP Feng Y Leng Y. Dipeptidyl peptidase 4 inhibitor sitagliptin protected against dextran sulfate sodium-induced experimental colitis by potentiating the action of GLP-2. Acta Pharmacol Sin. (2020) 41:1446–56. doi: 10.1038/s41401-020-0413-7, PMID: 32398684 PMC7656800

[255] Bang-Berthelsen CH Holm TL Pyke C Simonsen L Sokilde R Pociot F. GLP-1 induces barrier protective expression in Brunner’s glands and regulates colonic inflammation. Inflamm Bowel Dis. (2016) 22:2078–97. doi: 10.1097/MIB.0000000000000847, PMID: 27542128

[256] Kohli R Setchell KD Kirby M Myronovych A Ryan KK Ibrahim SH. A surgical model in male obese rats uncovers protective effects of bile acids post-bariatric surgery. Endocrinology. (2013) 154:2341–51. doi: 10.1210/en.2012-2069, PMID: 23592746 PMC3689286

[257] Chaudhari SN Luo JN Harris DA Aliakbarian H Yao L Paik D. A microbial metabolite remodels the gut-liver axis following bariatric surgery. Cell Host Microbe. (2021) 29:408–424.e7. doi: 10.1016/j.chom.2020.12.004, PMID: 33434516 PMC7954942

[258] Yao B He J Yin X Shi Y Wan J Tian Z. The protective effect of lithocholic acid on the intestinal epithelial barrier is mediated by the vitamin D receptor via a SIRT1/Nrf2 and NF-kappaB dependent mechanism in Caco-2 cells. Toxicol Lett. (2019) 316:109–18. doi: 10.1016/j.toxlet.2019.08.024, PMID: 31472180

[259] Wang W Zhao J Gui W Sun D Dai H Xiao L. Tauroursodeoxycholic acid inhibits intestinal inflammation and barrier disruption in mice with non-alcoholic fatty liver disease. Br J Pharmacol. (2018) 175:469–84. doi: 10.1111/bph.14095, PMID: 29139555 PMC5773980

[260] Argyrakopoulou G Konstantinidou SK Dalamaga M Kokkinos A. Nutritional deficiencies before and after bariatric surgery: prevention and treatment. Curr Nutr Rep. (2022 Jun) 11:95–101. doi: 10.1007/s13668-022-00400-9, PMID: 35174473

[261] Scheithauer TPM Davids M Winkelmeijer M Verdoes X Aydin O de Brauw M. Compensatory intestinal antibody response against pro-inflammatory microbiota after bariatric surgery. Gut Microbes. (2022) 14:2031696. doi: 10.1080/19490976.2022.2031696, PMID: 35130127 PMC8824225

